# The Problem of Purity in Moral Psychology

**DOI:** 10.1177/10888683221124741

**Published:** 2022-10-31

**Authors:** Kurt Gray, Nicholas DiMaggio, Chelsea Schein, Frank Kachanoff

**Affiliations:** 1 University of North Carolina at Chapel Hill, USA; 2 The Wharton School of the University of Pennsylvania, Philadelphia, USA; 3 Wilfrid Laurier University, Waterloo, Ontario, Canada

**Keywords:** moral psychology, political psychology, values, disgust, religion

## Abstract

**Academic Abstract:**

The idea of “purity” transformed moral psychology. Here, we provide the first systematic review of this concept. Although often discussed as one construct, we reveal ~9 understandings of purity, ranging from respecting God to not eating gross things. This striking heterogeneity arises because purity—unlike other moral constructs—is not understood by what it *is* but what it *isn’t*: obvious interpersonal harm. This poses many problems for moral psychology and explains why purity lacks convergent and divergent validity and why purity is confounded with politics, religion, weirdness, and perceived harm. Because purity is not a coherent construct, it cannot be a distinct basis of moral judgment or specially tied to disgust. Rather than a specific moral domain, purity is best understood as a loose set of themes in moral rhetoric. These themes are scaffolded on cultural understandings of harm—the broad, pluralistic harm outlined by the Theory of Dyadic Morality.

**Public Abstract:**

People are fascinated by morality—how do people make moral judgments and why do liberals and conservatives seem to frequently disagree? “Purity” is one moral concept often discussed when talking about morality—it has been suggested to capture moral differences across politics and to demonstrate the evolutionary roots of morality, especially the role of disgust in moral judgment. However, despite the many books and articles that mention purity, there is no systematic analysis of purity. Here, we review all existing academic articles focused on purity in morality. We find that purity is an especially messy concept that lacks scientific validity. Because it is so poorly defined and inconsistently measured, it should not be invoked to explain our moral minds or political differences.

Is it immoral for you to have consensual, loving, and safe sex with your sibling (Haidt, 2001)? What about selling your soul (Graham & Haidt, 2012), pouring urine over yourself (Chakroff et al., 2017), keeping an untidy living space (Clifford, Iyengar, et al., 2015), or purposefully wearing unmatched clothing (Horberg et al., 2009)? Although these acts are diverse, scholars suggest that they all tap the same construct: moral impurity—or more simply “purity” (Haidt, 2001; Haidt & Joseph, 2007). The topic of purity has seen an explosion of interest in moral psychology: in the 15 years from 2005 to 2019, 215 moral psychology articles have used the term “purity” in their titles and abstracts compared with only 3 articles in the previous 15 years (See Figure 1). The popularity of purity reflects an increased appreciation of moral pluralism and the rise of two influential theories—the social intuitionist model (Haidt, 2001) and Moral Foundations Theory (Haidt & Joseph, 2007)—that use the concept of purity to make their strongest claims. Despite the popularity and theoretical importance of purity, a pressing question remains unresolved—*what exactly is purity*?

**Figure 1.**
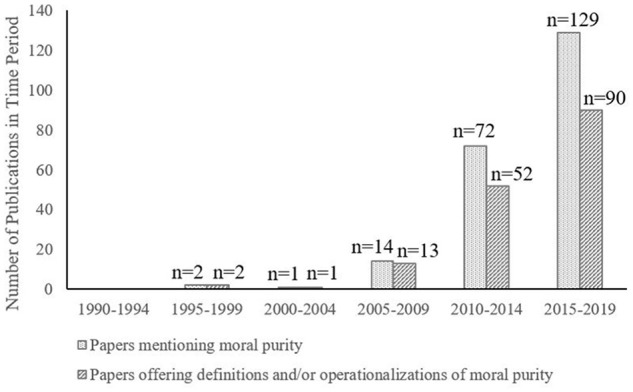
How common is purity in the field of moral psychology? *Note*. A number of publications per 5-year interval which (a) mentioned moral purity and (b) offered a definition or operationalization of purity related to morality. Data were generated from PSYCinfo. Keywords had to be present in the title, abstract, or subject.

Here we provide a systematic investigation of purity, exploring how this concept has been understood historically and how it is currently understood in the empirical studies and theories of moral psychology. We also evaluate four key purity-related claims in the field:

1. Purity holds an important place in moral rhetoric.
2. Purity shows cultural variation.
3. Purity is a coherent psychological construct.
4. Purity judgments are underlain by a distinct moral mechanism.

Our review reveals that despite strong evidence for claim one (moral rhetoric), there is ambiguous evidence for claim two (cultural variation), relatively little evidence for claim three (coherent construct), and no good evidence for claim four (distinct mechanism). As we show, the main problem facing claims involving purity is that purity itself is not well-defined: The field lacks a common and coherent understanding of purity. One could argue that this lack of coherence reflects mere variation across research teams, but we suggest the issue is more fundamental, reflecting a deeper truth about the nature of purity.

In contrast to most psychological constructs, which are defined “positively”—this construct is *x*—we suggest purity is defined “negatively”—this construct is *not y.* In the case of purity, this negative definition is “not interpersonal harm.” Being defined negatively allows for a very heterogeneous set of acts, qualities, and characteristics to count as purity. Although positively defined sets can sometimes be heterogeneous, negatively defined sets are necessarily more heterogeneous. As Bertrand Russel long ago noted, the set of “not *y*” (e.g., not cars, not cats, not even numbers) is much more varied than the set of “is *x*” (e.g., planes, dogs, odd numbers; Irvine & Deutsch, 1995). More succinctly, we suggest that purity is best understood as a “contra-chimera.” Purity is “contra” (defined as “in opposition or contrast to”) because it is understood as contrary to obvious interpersonal or “dyadic” harm (Schein & Gray, 2018) and as a chimera (defined as “a mixture of genetically different tissues”; Rogers, 2018) with a diverse set of characteristics and definitions.

In this article, we first review the historical development of the concept of purity. This review reveals substantial conceptual heterogeneity across history, which lays the groundwork for substantial conceptual heterogeneity across moral psychology. Second, we perform a systematic analysis of definitions and operationalizations of purity across all published papers from 1990 to 2019, which provides support for the idea that purity is a contra-chimera—a single name referring to a heterogenous set of understandings defined in contrast to obvious interpersonal harm. Third, we evaluate the four purity-relevant claims before providing recommendations for conducting future research on purity in moral psychology.

As we critically evaluate the idea of purity, we also critically evaluate the theories that lean heavily upon purity, notably the popular Moral Foundations Theory (Graham et al., 2013). Moral Foundations Theory assumes that purity is a coherent psychological concept and a separate cognitive moral mechanism. Our review reveals evidence against these two assumptions, suggesting instead that purity-related concerns—which are many and diverse—are grounded in culturally constructed perceptions of harm, as argued by the Theory of Dyadic Morality (Schein & Gray, 2018) and its recent extension, the Affective Harm Account (AHA; Gray et al., 2022).

## A Historical Overview of Moral Purity

### The Roots of Purity

Purity is an ancient concept. The Old Testament details numerous ways in which a person becomes ritually impure (*tah-meh*), including menstruation, childbirth, worshipping false idols, improper sexual relations, contact with death, consuming impure animals, and even violating clothing ordinances. Although some states of impurity (e.g., becoming impure through childbirth) are transient and easily washed away by a ritual bath, other violations are serious transgressions. According to Leviticus 20:18, having sex with a menstruating woman merits one of the strictest Biblical punishments—being exiled from the community. Beyond Judeo-Christian traditions, purity concerns are found across cultures. The Haida of British Columbia ritualistically “purify” themselves through fasting, abstinence from water, and purging with salt water to achieve success in war, hunting, and magic (Murdock, 1934). The Tikopians bathe before certain rites to avoid being dirty in the presence of God, and chiefs are required to refrain from magic and sorcery to preserve their physical and spiritual purity (Firth, 1960, 1970).

The historical importance of purity concerns is even broader if you consider the importance of taboos across cultures. In his investigation of the history of religions, F. B. Jevons (1896) cites “taboo” as a word having Polynesian roots, but points out its universality. Taboos are generally thought of as socially prohibited acts and practices related to holiness, cleanliness, and purity. These taboo acts center on safeguarding social purity around a variety of targets including the dead, the newborn, kings, criminals, food, personal property, and more. Taboos are especially dangerous because they do not require physical contact to harm people. In Samoa, for instance, the high priest was thought to cause trees to die by merely glancing in their direction, and in ancient Greece, there are tales of men who were driven insane by looking upon the image of Dionysus.

Other times, concerns about taboos prompt cleaning and purification rituals. In ancient Greece, worshipers were required to endure long purification rites before they could view sacred objects. Similarly, Peruvian chiefs would need to remove their sandals before contacting the soil around sacred idols to avoid infecting their sandals, which would render them unfit for daily use. As the famed anthropologist Sir James Frazer (1922) notes, “taboos act, so to say, as electrical insulators to preserve the spiritual force with which these persons are charged from suffering or inflicting harm by contact with the outer world” (p. XXI.1). The specific taboos that different cultures focus upon can vary, but they all revolve around the concept of purity, manifesting as sacred rites and prohibitions that urge people to avoid the apparent harmfulness of contacting the unclean (Radcliffe-Brown, 1939).

A review of the history of the term “purity” reveals a similarly wide-ranging concept. Early descriptions of purity—at least in the West—come from (Puritan) Christian sermons where the term referred to a perfect state of Godliness (Alleine, 1700). In the 1700s, purity became less Godly and more personal, with leading theologians warning their fellow Christians to resist “sordid passions or bestial desires” lest they lose their natural purity and become “lapsed and depraved” (de Charron & Stanhope, 1707; Tucker & St John Mildmay, 1805). Later still, Wisconsin’s first Mormon, Moses Smith (1861) used the term in a way that integrated conceptions of religious Godliness and bodily sin: “all that is impure must be rejected from the limitless purity of God in the future world” (p. 33).

As it evolved from its religious roots, the concept of purity expanded to apply to both biological and mental processes. In the 1800s, someone was seen to lack purity if they had bodily deformities or mental diseases. John Harvey Kellogg (1888), a medical doctor and cereal brand creator,^1^ closely linked purity to health, noting that impure acts such as lustful thoughts and unchaste actions not only “destroy the body” but also “dethrone the mind” and “ruin the soul.” Kellogg’s (1888) writing on purity demonstrates not only its growing perceived importance but also the diverse set of constructs he understood purity as being linked to: “persevering, conscientious efforts to comply with every requirement of health, purity, morality, and the laws of nature, will accomplish wonders in securing healthy children with good dispositions, brilliant intellects, and beautiful bodies” (in “A Source of Crime”). Here and elsewhere, purity was sometimes considered equivalent to physical health, to moral behavior, or to a basic capacity for self-control and self-restraint, upon which both physical health and moral behavior depend.

In the 1900s, discussions of purity became more psychological, with the rise of Freudian psychology. In what would come to be understood as the *Madonna-Whore Complex*, Freud lays out a form of psychic impotence based on the maladaptive split between “heavenly and earthly (or animal) love. Where such men love, they have no desire, and where they desire, they cannot love” (Freud, 1997). Here, Freud contrasts the noble pursuit of love with the base “return of the repressed” and goes on to illustrate how these “psychopathological” tendencies can develop. These psychoanalytic ideas helped spur the rise of purity-related psychological “folk” theories of the 1960s. Folk theories referred to lay beliefs around concepts such as “sexual purity” and “purity of thought” to describe values that varied across cultures as well as to characterize individual differences in personality (Agarwal, 1962; Parikh, 1964; White, 1962). Meanwhile, psychoanalysts continued to write about how unconscious beliefs about the sexual purity of women can impact psychosexual functioning and about how cultural artifacts like the presence of fire in various Judeo-Christian traditions come to symbolize purity (Beit-Hallahmi, 1976; Radomisli, 1967; Stephen, 1936).

Anthropological research in the 1960s also highlighted the salience of purity in non-Western cultures. In an influential review of purity, anthropologist Mary Douglas (1966) details the complex codes governing the behavior of Brahmin Indians, a priestly class within the Indian caste system. These codes include concerns over defiling one’s spirit through the consumption of unclean foods, contact with impure substances, or touching a person who is impure. These anthropological descriptions illustrated how conceptions of purity were culturally situated, which set the stage for moral psychologists to wonder whether purity was universally important to morality.

From the Puritans to Brahmins, purity has been linked to God and religion, bodily desires, sin (or its absence), and physical and mental health. This historical overview reveals that the term purity has been applied differently over time and is specific to cultural contexts. If there is any common theme, it is that purity is a morally loaded placeholder that allows writers to express diverse ideas of condemnation with one potent word. Despite this diversity—or perhaps because of it—the term purity was taken up by moral psychology, which we will soon see endorses each of these various historical definitions.

### Roots of Purity in Moral Psychology

Although the concept of purity is referenced in many religious and moral codes, it was widely ignored in early moral psychology research, which focused on principles of justice (Kohlberg, 1981) and causing and preventing physical harm (Turiel, 1983). The superstitious rituals and taboos that fell under the mantle of purity were discounted as matters of mere religious or social convention (Kohlberg, 1981; Turiel et al., 1987).

This narrow definition of morality was challenged, however, by cultural anthropologist Richard Shweder who conducted interviews on moral values in India and the United States (Shweder et al., 1987). He found that both Chicago natives and Brahmin Indians condemned unjustified physical violence, but only the Brahmin Indians condemned violations of food consumption norms, such as a son eating chicken shortly after his father’s death. On the one hand, these norms could be seen as safeguarding family members from harm because eating chicken was seen as blocking the father’s soul from reaching the afterlife (Schein & Gray, 2015). On the contrary, when justifying their moral position, the Brahmin Indians appealed to concerns traditionally tied to purity, such as polluting the soul and defiling the body (Shweder et al., 1987). From this finding, Shweder and colleagues (1997) concluded that morality concerns not only “ethics of autonomy” (individual rights) but also “ethics of divinity,” which are built on the notion of the self as “a spiritual entity connected to some sacred or natural order of things” (p. 138), and involves concepts of “sacred order, natural order, tradition, sanctity, sin, and pollution” (p. 138).

Shweder’s cross-cultural research sparked two developments in moral psychology. Most relevant to Shweder’s initial work is the cultural developmental theory of moral psychology (Jensen, 2015b), which treats divinity as one of three clusters of values (divinity, autonomy and community) revealed in moral reasoning and rhetoric. These values are understood not as mutually exclusive or competing but instead as co-existing, complementary, and even mutually reinforcing themes of discussion. In other words, rhetoric condemning the same act (e.g., gay marriage), can be framed in terms of divinity (gay marriage violates God’s will), community (gay marriage destroys fabric of society), or autonomy (gay marriage hurts children). The framework provided by cultural-development theory treats divinity not as a specific psychological mechanism or “domain” that is distinct from harm but rather as an important value often raised in discussions and justifications of moral judgment.

The other development inspired by Shweder’s work was the development of Moral Foundations Theory (MFT; Haidt & Joseph, 2007), which connects the cross-cultural diversity revealed by Shweder to modular evolutionary accounts of the mind. The most popular cultural-evolutionary account developed at the time of Shweder’s work was the theory of basic emotions (Ekman, 1992), which argues that evolution endowed humans with six distinct emotions, each of which serves separate functions and are grounded in separate psychological mechanisms. Echoing these basic emotional assumptions (Keltner & Haidt, 1999), Moral Foundations Theory argues that cross-cultural moral differences reflect the differential activation of five innate moral mechanisms (Haidt & Joseph, 2007): harm, fairness, loyalty, authority, and—most important for our purposes—purity.

The purity mechanism/foundation is thought to have initially evolved as a mechanism to prevent our ancestors from eating poisonous substances or touching disease-carrying corpses. MFT argues that because humans evolved in social group-based environments, humans’ aversion toward potential pathogens was coopted to become sensitive to a broader array of concerns, including more symbolic “impurity” violations of the natural order, personal sanctity, and God’s will (Haidt, 2012). Testable predictions about the purity mechanism/foundation argue that it is specifically connected to the “basic emotion” of disgust (Horberg et al., 2009), and functionally distinct from concerns about harm (Haidt, 2012; Haidt & Hersh, 2001).

### Purity’s Contribution to Moral Psychology

It is not an overstatement to claim that the concept of purity revolutionized the study of morality. As we now briefly review, purity research did three main things. First, it prompted a shift from models heralding a singular moral truth (Kohlberg, 1969), to the widespread embrace of cultural diversity and moral pluralism. Second, it accelerated a shift from rationalist models of moral judgment to the embrace of intuition (Haidt, 2001), pulling moral psychology further away from its roots in moral philosophy. Third, purity research helped to show how morality expands beyond concerns of concrete physical and emotional harm.

#### Cultural diversity

Kohlberg’s (1969) cognitivist account of moral development postulated the existence of an ultimate, universal moral truth, centered around abstract Kantian notions of impartial justice. According to Kohlberg, there is a clear endpoint of moral development—to become a rational, fully developed, mature moral thinker (i.e., like Kant)—and moral disagreements exist only because some people plateau at more rudimentary moral stages. This universalist account of moral psychology downgraded individuals who spoke in a “different moral voice,” including women who prioritized care and commitment to loved ones over abstract impartial moral norms (Gilligan, 1993).

By revealing that the Brahmin Indians morally condemn actions related to sex, religion, the body, and food, purity research brought moral diversity to the forefront of moral psychology. This research also argued that non-Western cultures—despite not being familiar with Kant—nevertheless still have fully developed moral codes, even if they are not based on universal and impartial moral values, and even if they conflict with Western views of justice (Miller & Bersoff, 1992; Rai & Fiske, 2011; Shweder & Haidt, 1994). In contrast to Kohlberg, Shweder and colleagues argued that non-Western moral concerns do not reflect a more “primitive” moral development but instead the existence of genuine—and equally valid—moral beliefs that extend beyond Western philosophical concepts (Shweder, 1984).

Thanks to purity research, the field of moral psychology has largely accepted the existence and importance of moral diversity and the genuineness of non-Western moral concerns. It is still an open question about what causes these cultural variations in morality and whether it requires “deep” differences in psychological mechanisms. Elliot Turiel argued for a harm-based view of moral judgment but also argued that different “cultural assumptions” about what is harmful and who can be harmed can explain cultural variation in moral judgment (Turiel et al., 1987). MFT rejected these claims and argued that cultural differences require a diverse set of distinct evolutionary-psychological modules (Graham et al., 2013). Recent work on the Theory of Dyadic Morality (TDM; Schein & Gray, 2018) has put social cognitive evidence behind the claims of Turiel, revealing how a broader understanding of harm can account for cultural diversity without requiring a set of distinct moral modules. Regardless of how theories of moral cognition explain moral diversity, all tenable theories must at least acknowledge moral diversity.

#### Intuitionism

In addition to broadening the scope of morality, research on purity directly challenged the Kohlbergian claim that moral judgments are a product of careful reasoning about harm (Kohlberg, 1969). In what is likely the best-known moral psychology demonstration, Haidt asked participants why it is wrong for two siblings to have consensual, loving, safe sex. This vignette was seen as a purity violation rather than a harm violation because it was “carefully written to be harmless” (Haidt et al., 2000, p. 5). Each time participants appealed to the potential rationalist, harm-based reasons (e.g., the siblings might have deformed children), the experimenter argued that those reasons were invalid (e.g., potential children are not an issue because contraceptives were used). Eventually, once all the reasons offered by participants had been dismissed, participants stopped offering additional reasons, a phenomenon labeled “moral dumbfounding” (Haidt et al., 2000).

This speechlessness was interpreted as evidence of the primacy of moral intuitions over moral reasons (Haidt, 2001). Haidt argued that if people cannot articulate valid reasons for their moral judgments, then perhaps reasoning was never the driving force behind these condemnations. There are a number of critiques of the “moral dumbfounding” paradigm (Royzman et al., 2015) and doubts about the perceived harmlessness of brother–sister incest (Schein & Gray, 2018). For example, it is unclear why perceptions of harm cannot be intuitive, just like moral judgment. Nevertheless, it is certainly true that considering incest feels somehow different than considering whether a husband should steal a drug to help his ailing wife—a scenario that Kohlberg used to highlight the importance of reasoning (Kohlberg, 1981). Of course, one difference is that the incest scenario is not an obvious “dilemma,” as participants uniformly view incest as immoral. But Haidt and others highlighted another difference, which is that incest elicits disgust in a way that questions about rights and duties do not.

The descriptive link between disgust and moral condemnation in purity scenarios spurred the development of the social intuitionist model, which argued that all moral judgments are fundamentally grounded in affect-based intuitions rather than calculated reasons. Questions still arise about the possible role of moral reasoning in shaping intuitions (Pizarro & Bloom, 2003), but purity violations made clear that intuition plays an important role in moral judgment. That many moral psychological theories and paradigms (e.g., work on moral dilemmas) consider the role of emotion and intuition in moral judgment (e.g., Greene, 2009; Greene et al., 2001) can be attributed in part to work on purity. Even a recent extension of the Theory of Dyadic Morality—the Affective Harm Account (Gray et al., 2022)—explicitly acknowledges the role of affect in moral judgment, especially for the ambiguously harmful and often-bizarre scenarios used to represent impurity.

#### Beyond harm

Historical discussions of impurity often highlighted how impure acts could lead to harm. Kellogg suggested that impure foods, thoughts, and behaviors could all undermine physical health and the functioning of society (Kellogg, 1888). Brahmin Indians believe that impure acts following someone’s death could result in that person being forever condemned to purgatory (Shweder et al., 1997). However, to the eyes of Western moral psychologists, these acts seemed objectively harmless, and this apparent harmlessness was a force behind both moral pluralism and moral intuitionism. If moral psychology, under the leadership of Kohlberg, focused on how WEIRD men (Western, educated, industrialized, rich, democratic; Gilligan, 1993) reasoned about harm, then the new moral psychology (Haidt, 2007) explored how more diverse groups intuitively reacted to considerations beyond obvious physical/emotional harm.

As mentioned earlier, the most popular, canonical, and first acts used to represent impurity were “carefully written to be harmless” (Haidt et al., 2000, p. 5). These scenarios of loving incest, masturbation with unwitting pets, and necrophilia revealed that acts without obvious interpersonal harm were nevertheless seen as immoral. Judgments of these acts not only helped inspire intuitionism and pluralism but also led to the popular Moral Foundations Theory (Graham et al., 2013), which restricts the role of harm to only one of five hypothesized moral mechanisms. Although research reveals that interpersonal harm occupies at least 95% of people’s everyday moral concerns (Hofmann et al., 2014), it is true that moral psychologists had neglected these bizarre sexual scenarios. They had also neglected a number of immoral acts often discussed by philosophers, such as harmless lies (Kant, 1797), breaking promises to the dead (Narveson, 1963), and justifying the torture of children (Le Guin, 1973).

Out of all potential moral concerns, purity is understood to be the most “harmless,” but there is increasing doubt about the harmlessness of impure acts—at least in terms of perception. Studies find that people robustly perceive harm in active immorality, whether they involve “harmless” incest (Gray & Keeney, 2015a; Royzman et al., 2015), entertaining sacrilegious thoughts (Schein et al., 2016), or other bizarre sexual behaviors (Gray & Keeney, 2015a). Correlations between ratings of impurity and harm are so high (*r*s > .86; Gray & Keeney, 2015b) that they seem not to be distinct constructs. Of course, one could argue about whether we should be prioritizing the judgments of participants (who see impurity as harmful) versus researchers (who see impurity as harmless), but moral psychology—with its roots in anthropology—has long emphasized privileging the intuitions of participants (Haidt et al., 2000). Although participants intuitively perceive harm in purity violations, we do acknowledge that these violations are less obviously harmful (and more affectively evocative) than interpersonal harm (Gray et al., 2022). Immoral acts involving interpersonal harm may be the most universal, common, and consequential (Hofmann et al., 2014), but it is important for moral psychology to understand how people react to all moral acts, not just the most common and consequential of them—as work on purity demonstrates.

### Purity Evolves Into Moral Mechanism

Purity has powerfully shaped modern moral psychology. In the broadest sense, the impact of purity can be understood as the infusion of anthropology into a subfield of psychology dominated by philosophy. Purity showed us that morality may not be the invariant, universal, and reason-based domain it was long believed to be. Rather, morality hinges upon cultural understandings that vary across time and place and upon intuitions that have one foot in our evolutionary past. However, it is worth considering how the idea of purity changed as it moved from the notebooks of ethnographers to the labs of psychologists.

In its first documentation in anthropology (Douglas, 1966), purity was used as an umbrella term that encompassed many different ideas, including respecting divinity, keeping bodily sanctity, guarding against spiritual degradation, and maintaining the integrity of both food and rituals. More modern cross-cultural anthropological research also understands purity as a cluster of values present in moral discourse (see also Jensen, 2015b) that can be meaningfully applied to any act by anyone passing moral judgment (DiBianca Fasoli, 2018). The work of Shweder saw purity as a direct contrast to values of autonomy—of individual rights—which traditional moral psychology long saw as dominating moral judgment. But Shweder et al. (1987, 1997) also acknowledged that the construct of purity contained a heterogeneous set of acts and subvalues that shaped cultural norms.

In its leap to moral psychology, purity became reified, transformed from a loose constellation of values raised in conversation to a concrete psychological mechanism. This transformation was difficult to avoid; although psychology deals in descriptive differences, modern social psychology is grounded in the assumptions of “social cognition” which focuses on revealing the cognitive mechanism underlying psychological phenomena. In the case of purity, this cognitive reification was clearly apparent, transforming a heterogeneous collection of cultural values into an evolutionary-cognitive module, arguably the most concrete of psychological mechanisms. MFT argues that purity is a distinct cognitive mechanism triggered by one specific type of act (i.e., a purity violation) grounded in one specific emotion (i.e., disgust; Graham et al., 2013; Haidt et al., 1993; Horberg et al., 2009; Rozin et al., 1999). MFT also argues that some groups (e.g., conservatives) have a more developed purity mechanism than other groups (e.g., liberals), which results in different moral judgments, different voting behavior, and even different ways of speaking (Haidt, 2012).

Given this cognitive consolidation of purity into a concrete psychological mechanism, we must ask whether our descriptive understanding of purity has also been consolidated. When moral psychology refers to a “purity foundation” triggered by a “purity violation” what do we mean by “purity?” Is purity a *coherent concept*—something that is unitary and distinct from other concepts? To explore this idea, we first start with the question of whether there is a coherent *definition* of the concept of purity—at least as coherent as any other moral psychology concept.

### Searching for a Psychological Definition of Purity

How does moral psychology define purity? Before answering this question, we first consider whether we even need a definition of purity. Some may argue that trying to define concepts only distracts from doing science. There is no clear definition of “life” (e.g., Koshland, 2002; Macklem & Seely, 2010) but the scientists who study living systems seem to get along just fine. Scholars disagree about whether viruses are alive (Koshland, 2002), but viruses can still be observed under a microscope, have their genetic material manipulated, and be targets for vaccines.

Precise definitions may be distracting when studying the physical world, but we suggest that they are important when studying mental processes because psychological concepts (e.g., purity) are fundamentally different from biological concepts. A psychological concept is a “private-event” construct that *cannot* be directly observed or quantified independently of how the researcher defines and operationalizes it (Moore, 2009; Watson, 1913). Regardless of whether a virus is truly alive, these physical entities and processes will always be observable in the same way. Yet private-event constructs are only what researchers make of them: If two different researchers have different conceptions about the nature of private-event construct, then their measurement of those constructs will also be very different as will their conclusions. More concretely, if two researchers define and operationalize purity differently, then can we really say that they are studying the same construct?

Having a clear definition of purity is not only essential for isolating psychological processes, but it is also important for society. Psychologists have used the concept of purity to explain the political divides surrounding hot-button issues such as debates about vaccination (Amin et al., 2017) and gay marriage (Inbar et al., 2009, 2012). These purity-based explanations have given rise to recommending interventions to bridge partisan divides, such as using purity-based language to motivate conservatives to care more about environmental issues (Feinberg & Willer, 2013). Crafting effective and feasible interventions for social problems requires isolating and targeting the psychological mechanism behind those problems (Walton & Wilson, 2018). If the efficacy of an intervention relies on targeting the psychological mechanisms of purity, we must know what purity is.

In a perfect world, a construct could be defined based on a set of necessary and sufficient features. But as Wittgenstien realized when trying to define the concept of a “game,” strict definitions are elusive even for simple constructs (see Kenny, 1973). Nevertheless, it is possible to generally define a construct such that it captures much of the key features; as proof, one needs to look only to the existence of dictionaries. Consider birds—although some birds do not fly, they generally do fly, and at least we can say that they generally have wings. Likewise, although there is one mammal that lays eggs (the platypus), we can say that mammals are generally furry warm-blooded animals that give live birth and nurse their young. Notice that these definitions outline concrete and specific properties that we should expect to find in exemplars—even if those properties are only found probabilistically.

It should be possible to generally define purity at least as well as other moral psychological concepts. Consider harm. Despite variability in what people consider harmful, and social “concept creep” in the nature of harm (Campbell & Manning, 2018; Haslam, 2016), it is relatively simple to specify the core defining features of harm: someone causing physical or psychological damage to another. In fact, harm is concrete enough that it can be specified through an equation, with the Theory of Dyadic Morality defining harm as “A→P,” an intentional agent causing damage to a vulnerable patient (Schein & Gray, 2018). Of course, people may disagree about whether someone is truly intentional or vulnerable, and how much some kinds of acts cause psychological damage (e.g., microaggressions; Lilienfeld, 2017; Lui & Quezada, 2019; Ong & Burrow, 2017). This disagreement, however, is about the fringes of the concept; there is widespread agreement about clear exemplars of the construct. In fact, although Moral Foundations Theory and the Theory of Dyadic Morality disagree about the nature of the moral mind, both fundamentally agree on canonical acts of harm: child abuse and emotional cruelty (Graham et al., 2013; Schein & Gray, 2018). As we will see, there is no such clear agreement with purity.

A final point to make about the nature of well-defined concepts is that definitions must not be tautological. Definitions of harm are not self-referential; they can be broken down into smaller elements that are not themselves those concepts. The elements of morally relevant harm can each exist without referring back to the overarching concept of morally relevant harm, including intentional agency (e.g., being an author), causing an action (e.g., rowing a boat), and physically suffering (e.g., drop a book on your foot). To define the purity of moral psychology, it should also be possible to isolate the defining components of “moral purity” without invoking purity itself. As we will see, definitions of purity seem either to invoke synonyms of purity (e.g., “defilement,” “taint”), immorality in general, or focus on what purity *isn’t*.

## A Hypothesis About Purity: The Contra-Chimera Definition

In the next section, we systematically review the moral psychology literature, examining how researchers have understood this important topic. But first, we outline a hypothesis for how purity is understood: as a “contra-chimera.” This hypothesis involves two complementary claims, the “contra” and the “chimera.”

### Purity as *Contra*-Harm

Purity was instrumental in broadening how moral psychology understood morality, making researchers consider the immorality of acts beyond direct physical harm, especially ostensibly harmless violations related to food and sex (Haidt, 2001; Haidt et al., 1997, 2000). Because of its historical role as a foil to harm, we hypothesize that purity remains understood as a set of acts that are not obviously harmful. The classic act of consensual brother–sister incest was explicitly created to be objectively harmless (Haidt et al., 2000), and the purity violations of Brahmin Indians also captured attention because of their apparent harmlessness to Westerners (Shweder et al., 1987). Given that many theoretical claims in modern moral psychology rely on the presumed distinctness of purity from harm (Graham et al., 2009, 2013), we suggest that purity will often be defined as contrary (i.e., contra) to harm. More technically, purity will be defined as a negative set rather than a positive set.

Philosophers including Georg Cantor, Gottlob Frege, and Bertrand Russell developed the idea of naïve sets in mathematics, which are arbitrary collections of entities (see, Bagaria, 2014). Positive sets are defined via members sharing a common feature, such as the set of all barbers, or the set of all animals that are house cats (Friedman, 2003). In contrast, negative sets are defined via members sharing a *lack* of a common feature, such as the set of all people who are not barbers, or the set of all animals that are not cats. Many moral concepts, such as harm, fairness, and loyalty, are positive sets because they each share a set of key features, whereas we suggest that purity is a negative set, unified by the nature of *not* being harm.

Although negative sets do provide a commonality between members, they are less scientifically useful than positive sets. One problem is that it is difficult to draw inferences from negative sets because negative sets are much larger than positive sets. For the positive set of “barbers,” it is true that barbers can differ on many features, but there is even more variation among all people who are “non-barbers.” There are many varieties of cats, but there are even more “non-cats.” If purity is defined on the basis of not-harm, then the number of potentially impure acts is extremely high and extremely diverse.^2^

It is difficult to draw inferences about psychological concepts in the first place, but only one inference can be made about negative sets: that the key missing feature (e.g., harm) is not necessary for defining it (e.g., purity). One can see evidence for this inference in moral psychology, where researchers have used “harmless” purity violations to argue that harm is not necessary for moral condemnation (Haidt, 2001; Haidt et al., 2000).

A related problem with a negative definition is that falsification is difficult. With a positive set, there is a clear set of acts that lie at the “center” of the set—the most canonical members. If these central members fail to act as expected, then one can confidently say that the set does not act as expected. For example, you could define the set of mammals as “egg-laying animals,” but this claim is not true when examining canonical mammals such as bears, lions, and squirrels that lie at the “center” of the concept. Accordingly, we would say that the idea of mammals as egg-laying has been generally falsified, even if it is true of platypuses.

But now consider a similar claim for negative sets. Imagine arguing that the set of animals who are *not* mammals are egg-laying animals. This claim is now much harder to disprove because this negative set is so much bigger and heterogeneous. This claim could be true or not depending on what you select: true for birds and lizards and false for worms and sponges. If purity is defined as contra-harm, then one could always point to a new set of acts to deflect theoretical criticism, making it hard to falsify theories related to purity.

### Purity as a *Chimera*

The infiniteness of negative sets, and their lack of a clear conceptual “center,” means that negative sets typically include many very different members. Consider again the difference between the positive set of animals that “are cats” and the negative set of animals that “are not cats.” The positive set of “house cats” is itself a single coherent animal or at least as coherent as a single mental construct tends to be. This conceptual coherence makes it possible for different people to draw a cat and have their drawing look similar to each other and for people to chuckle at cat cartoons that make fun of their shared natures. Conversely, the set of not-cats is not a coherent animal. If one drew a “not cat,” it would not be recognizable by other people. Instead, it would be seen as belonging to some arbitrary positive set, whether dogs, monkeys, or Himalayan golden-backed three-toed woodpeckers.

If one approached a negative set with the same expectations of a positive set—namely that there is a single canonical understanding—the result would be a chimera. The biological definition of a chimera is “an organism or tissue that contains at least two different sets of DNA, most often originating from the fusion of many different zygotes (fertilized eggs)” (Rogers, 2018). Our psychological definition of chimera is when a concept is thought to be a single thing (i.e., it is referred to by the single name of “purity”) but has many different understandings as revealed by heterogeneous definitions or operationalizations. Mirroring the heterogeneity in understandings of “purity” across history and culture, we suggest that purity is a chimera possessing many different scientific understandings.

Before moving to our systematic analysis of whether purity is a contra-chimera, we address two potential criticisms of our contra-chimera argument. The first is factual: Our contention that purity is a contra-chimera is invalid because there obviously *is* a clear center to impurity—the classic case of sibling sex (Haidt et al., 2000). While most agree that this act counts as impurity, one cannot build a science upon a single operationalization in a single study by a single set of researchers, lest we have moral psychology so narrow that it only applies to one thought experiment. For purity to have the broad importance it is often ascribed, it is necessary to examine other operationalizations in other studies by other researchers. Science is a collective enterprise. Even if researchers generally agree that consensual sibling sex is impure, science develops through a cycle of connecting abstract theories with concrete operationalizations. In the case of purity, we need to connect sibling sex back to definitions, and we need to know why this act is impure.

A second potential criticism of our contention that the nature of purity is a contra-chimera is “so what?” One may also argue that limiting purity to a single positive-set definition with a common set of acts is too restrictive and that purity shouldn’t be held to that standard. We again note that scientific inference and falsifiability hinge on the ability to positively define a coherent construct. In addition, other moral psychology definitions—not only harm, but also loyalty, industriousness, tradition, and security—are not contra-chimera, being defined by coherent positive definitions and being operationalized by a set of qualitatively similar acts.

It is true that anthropological work treats purity as a kind of meta-value through which various values are connected in moral rhetoric (Jensen, 2015b; Shweder et al., 1987, 1997). This could make purity a kind of “meta-positive-set” populated with many values, existing as a menagerie of distinct moral concerns rather than a single chimeric creature. This possibility is certainly defensible but only if purity is understood as a loose collection of discourse themes as in the work of Jensen (2015b) and DiBianca Fasoli (2018). In contrast, modern moral psychology argues that purity is a single cognitive mechanism, a mechanism that is functionally the same as other moral mechanisms that “detect” and are “triggered” by coherently defined concepts such as harm and fairness (Graham et al., 2013). As such, it is imperative to ask precisely what moral content is thought to “trigger” purity judgments?

## Reviewing How Purity Is Defined and Operationalized

To examine how purity is understood in moral psychology, we retrieved all papers that contained the word “purity” either in the title, abstract, or text, and which were published between 1990 and 2019 in any peer-reviewed journal contained within the PsycInfo archive. Our search yielded a final corpus of 158 papers which defined moral purity in the main text, with 135 of these papers operationalizing/measuring moral purity.^3^

With this corpus, we systematically reviewed and coded how purity was defined. We also coded how purity was operationalized, as an implicit examination of how purity was understood. Not only are operationalizations the concrete basis of psychological studies, but they can often depart substantially from explicit definitions in the introduction, such as being much narrower. Consider how researchers discuss the richness of “love,” and then operationalize it as questionnaire ratings (Rubin, 1970), or discuss the importance of “cooperation” and then operationalize it as the prisoner’s dilemma (Axelrod, 1980), or highlight the nuances of “race” and then operationalize it as Black versus White names (Bertrand & Mullainathan, 2004). This is not a criticism as any one study must examine a concrete manifestation of a concept, but it is informative to look at operationalizations and evaluate them for consistency—both consistency with definitions within the same paper and with other operationalizations across different papers.

We systematically reviewed both definitions and operationalizations. For each, we identified whether it was (a) stand-alone (a positive definition, not understanding purity in contrast to harm) or contra-harm (a negative definition, understanding purity in contrast to harm—whether explicitly or implicitly) and (b) a single, coherent understanding (purity is X) or a chimeric understanding (purity is X, Y, and Z). Next, we looked across the various definitions and operationalization and—using history as a guide—identified the various “understandings” of purity. Finally, we used these understandings to quantify the variation in purity understandings both across papers and within papers (i.e., do operationalizations of purity match the definitions?) For the full coding table and other supplemental materials, see https://osf.io/quxt9/?view_only=ad359a04c56b4320889ac10b9235389b.

### Definitions of Purity

We retrieved 158 articles that offered a definition of purity. Twenty-seven additional articles retrieved in our initial search cited MFT and utilized the Moral Foundations Questionnaire (which touches on purity) but did not offer their own purity definition. These articles were not included because researchers may not endorse the underlying theoretical assumptions of that instrument. For each definition, we examined how much it was understood as contra-harm and chimeric. We recognize that there is always the potential for bias when researchers code materials, which is why the full text of all 158 definitions is available in the supplement.

#### Contra-harm?

We classified definitions of purity into three categories: “explicitly contra-harm,” “implicitly contra-harm,” or “stand-alone.” A definition was coded as “explicitly contra-harm” if it outright described purity violations as moral violations that did not involve harm. For example, the definition offered by Graham et al. (2009) describes “issues related to food, sex, clothing, prayer, and gender roles as moral issues, *even when they involve no harm to any person*” (p.1030; italics added for emphasis). Similarly, the definition of purity offered by Haidt (2007) describes purity as “intuitions about bodily and spiritual purity and the importance of living in a sanctified rather than a carnal way” (p. 1001) and states that “morality is about *more than harm and fairness*” (p. 998; italics added for emphasis). Thus, this definition explicitly refers to purity as something other than just harm. Likewise, Vasquez and colleagues (2001) described purity violations saying: “Some breaches did not violate rights or involve physical harm, but were instead disrespectful, analogous to Community, and disgusting, analogous to Divinity” (p. 96). Again, purity violations are explicitly described as not causing harm (note that Vasquez and colleagues explicitly state that purity and divinity are used interchangeably; pg 98.).

A definition was coded as “implicitly contra-harm” if it directly contrasted purity violations with harm violations without directly stating that purity violations were different from or absent of harm. These definitions typically come from more recent purity research that relies upon earlier works that explicitly contrast purity and harm as their theoretical foundation. For instance, McAdams and colleagues (2008) described purity as “corruption, contamination, defilement, imperfection, or other aspects of human life that deviate from that which is sacred, pure, or perfect” and then contrasted these to harm violations defined as “concern for protecting people from pain, injury, abuse, poverty, or some other form of physical or psychological suffering” (p. 986). Furthermore, McAdams uses Haidt’s (2007) assertion of moral intuitions as “evolved mechanism[s] or learning module[s]” as a starting point for their theoretical understanding of these moral concerns (p. 985). Although McAdams and colleagues did not explicitly describe purity as contra-harm, by building off previous literature which does and directly juxtaposing their definition of purity violations in contrast to a definition of harmful violations, they imply that purity is distinct from and a foil to harm. Similarly, Glenn and colleagues (2009) defined purity as “representing the moral ideal of living in an elevated, noble, and less carnal way, based on intuitions about divinity, feelings of moral disgust, and purity of body, mind and soul” and harm as “representing concerns about violence and the suffering of others, including compassion and care” (p. 386). Again, although Glenn did not explicitly state that purity violations are contra-harm, they imply this by juxtaposing purity violations to harm violations and citing works that support “distinct moral foundations” (p. 386) as the theoretical starting point (i.e., Graham et al., 2009).

Finally, a definition was coded as “stand-alone” if it neither explicitly described purity violations as distinct from harm nor implicitly implied such a distinction by juxtaposing purity violations with harm violations. For example, Helzer and Pizarro (2011) defined purity violations such as “viewing pornography, littering, and using drugs” (p. 517) and make no allusions to these as distinct or modular psychological mechanisms. Here, purity is never presented as a foil to harm nor is it juxtaposed to harm. Similarly, Preston and Ritter (2012) describe acts of purity as “protecting the individual from potential pathogens” (p. 1365). Again, in their definition, Preston and Ritter neither present purity as a foil to harm nor juxtapose a purity definition with a harm definition.

#### Chimera

Definitions of purity were classified as either a “chimera definition”—combining more than one understanding of purity—or a “single definition” that featured only one understanding of purity (i.e., purity is “X”). As an example of a single definition, Bastian and colleagues (2015) define purity as “the absence of immoral and therefore dangerous thoughts” (p. 1070). Here, impurity is limited to mental states involving one clear thing: immoral thoughts. Similarly, Berman and Small (2018) defined purity as “the extent to which someone lacks temptation to sin” (p. 220). Again, we see that this definition limits purity to only a lack of temptation rather than a set of qualitatively distinct phenomena.

In contrast, chimeric definitions define purity as a combination of at least two or more distinct understandings of purity—purity is “X,” “Y,” or “Z”—in the same way that a biologic chimera is a combination of at least two distinct genetic components. For example, McAdams and colleagues (2008) defined purity as consisting of multiple understandings when they described: “the body and certain aspects of life are sacred; cleanliness and health, as well as their derivatives of chastity and piety, are all good; dirt, pollution, contamination, and the associated character traits—lust, gluttony, and greed—are all bad”; (p. 985). From this definition, purity can involve multiple distinct behaviors or mental states. It refers simultaneously to maintaining sexual chastity (e.g., “lust,” “chastity”), self-control (e.g., “greed” and “gluttony”), and pathogen avoidance (e.g., “dirt,” “cleanliness,” “contamination,” “pollution”). Similarly, Koleva and colleagues (2012) defined purity as “based on the emotion of disgust in response to biological contaminants (e.g., feces or rotten food), and to various social contaminants like spiritual corruption, or the inability to control one’s base impulses” (p. 185). Here, purity again involves several distinct behaviors or mental states: It pertains to pathogen avoidance (“biological contaminants”), spiritual integrity (“social contaminants like spiritual corruption”), or self-control (“inability to control one’s base impulses”). Notably, across these two definitions, although some of the understandings of purity represented in the definition overlap (both allude to self-control and pathogen-avoidance), other understandings differ (Koleva and colleagues focus on spiritual integrity while McAdams and colleagues focus more on sexual chastity). Moreover, even when describing common understandings, the examples used to illustrate these understandings often vary across papers, speaking to the variability of chimeric definitions.

#### Results

In Table 1, we summarize the results of this analysis and provide example definitions for each of the 6 cells when crossing Contra (explicit, implicit, stand-alone) and Chimera (single, chimera). Supporting the contra-harm hypothesis, the majority of purity definitions either explicitly (15.8%) or implicitly (60.1%) defined moral purity as immoral behaviors that did not involve harm. In other words, <25% of definitions conceptualized purity without contrasting it with harm. Supporting the chimera hypothesis, the vast majority (96.2%) of definitions of purity described multiple understandings/behaviors/mental/physical states. Finally, it was notable that 100% of explicitly contra-harm definitions were also chimeras, supporting our claim that negative-set (contra) definitions give way to greater heterogeneity in how a construct is defined. We next investigated whether operationalizations of purity also revealed evidence of the contra-chimera hypothesis.

**Table 1.** Different Types of Definitions of Purity.

	Single definition (*n* = 6; 3.8%)	Chimera definition (*n* = 152; 96.2%)
Explicitly contra-harm (*n*=25; 15.8%)	**0 definitions**	**25 definitions** *“Research in India, Brazil, and the United States, for example, has found that people who are less Westernized treat many issues related to food, sex, clothing, prayer, and gender roles as moral issues [. . .]*, ***even when they involve no harm to any person***.*”* (p. 1030) *“And lastly, virtues of purity and sanctity that play such a large role in religious laws* ***[respect for God]*** *matched writings on the evolution of disgust* ***[disgust]*** *and contamination sensitivity* ***[pathogen avoidance]*** *[. . .]. Practices related to purity and pollution must be understood as serving more than hygienic functions. Such practices also serve social functions, including marking off the group’s cultural boundaries [. . .] and suppressing the selfishness* ***[self-control]*** *often associated with humanity’s carnal nature (e.g., lust* ***[chastity/sexual taboos]***, *hunger, material greed) by cultivating a more spiritual mindset* ***[spiritual integrity****]”* (Graham et al., 2009, p. 1031)
Implicitly contra-harm (*n*= 95; 60.1%)	**3 definitions** *“humans have evolved these binding foundations as a way to . . . rise above their base urges and exercise self-control (purity).* ***[self-control]****”* (Napier & Luguri, 2013, p. 754)	**92 definitions** *“* ***(The ethics of Autonomy) Individual freedom/rights violations. In these cases, an action is wrong because it directly hurts another person****. . . To decide if an action is wrong, you think about things like harm. . .* ***(The ethics of Divinity)*** *Divinity/purity violations. In these cases a person disrespects the sacredness of God* ***[respect for God]***, *or causes impurity or degradation to himself/herself, or to others. To decide if an action is wrong, you think about things like sin, the natural order of things* ***[natural order]***, *sanctity, and the protection of the soul or the world from degradation and spiritual defilement.* ***[spiritual integrity]****”* (Rozin et al., 1999, p. 576)
Stand-alone (*n*=38; 24.1%)	**3 definitions** *“mental purity (i.e., the absence of immoral and therefore dangerous thoughts)* ***[mental purity]****”* (Bastian et al., 2015, p. 1070)	**35 definitions** *“Research on the correspondence between physical and moral purity has speculated that people are predisposed to use categories that are based on bodily experience (such as clean versus dirty)* ***[pathogen avoidance]*** *to construct complex social categories (such as moral versus immoral)* ***[general immorality]***. *For example, in English, words such as “clean” and “pure” describe both physical and moral states (e.g., he has a clean record). Likewise, the Mandarin phrase “a pair of dirty hands” refers to a person who steals. The association between bodily and moral purity may be based not only in cognition, but in emotion as well. As an example, disgust represents an emotion that is experienced in both physical and moral domains.”* (Zhong & Liljenquist, 2006, p. 1451)

*Note*. Shown examples are taken from the most highly cited definition available within the category cell.

### Operationalizations of Purity

Our corpus of 158 papers contained 135 operationalizations of moral purity. We applied a similar coding strategy to operationalizations as we did the conceptual definitions of purity to test the contra-chimera hypothesis.

#### Contra-harm?

Operationalizations of purity were similarly coded as “explicitly contra-harm,” “implicitly contra-harm,” or “stand-alone.” An operationalization was coded as “explicitly contra-harm” if they included vignettes or questions which explicitly stated that the behavior was not harmful. For instance, The Moral Foundations Questionnaire (Graham et al., 2009) measures purity by asking people whether they condemn acts that are described as disgusting, but explicitly not harmful: “It bothers me when people do something disgusting, even if no one is harmed” (item 1, p.1044). Some vignettes used to describe purity violations also explicitly describe the violation as harmless. For example, to assess moral condemnation of a purity violation, Sabo and Giner-Sorolla (2017) presented participants with vignettes including “consensual sibling incest” (p. 136). Here, the behavior in question is explicitly described as not harmful when it states “Experiments 1 through 4 define purity code violations as abnormal acts that involve an immoral use of one’s body without harming specific others” (p. 135).

Operationalizations of purity were coded as “implicitly contra-harm” if they contrasted moral transgressions that explicitly involved harm versus similar transgressions which did not explicitly involve harm. For instance, Rottman and Young (2019) operationalized a purity violation using the vignette, “A person has intercourse with a goat,” and in direct juxtaposition, operationalized a harm violation using the vignette, “A person starves a goat.” Piazza and colleagues (2013) had participants rate both a “harm transgression (a neighbor had kicked their pet dog)” or “an equivalent purity transgression (a neighbor had cooked and eaten their pet dog after it died of natural causes)” (p. 709).

An operationalization of purity was coded as “stand-alone” when the items or vignettes used to assess the purity violation neither explicitly described the violation in question as “harmless” nor did it juxtapose similar but different scale items to contrast purity violations from harm violations. For example, Helzer and Pizarro (2011) measured purity with items such as “While house sitting for his grandmother, a man and his girlfriend have sex on his grandmother’s bed” or “After a late-term miscarriage, a woman asks her doctors to take a picture of her cradling the miscarried fetus” (p. 519). Here, the violations in question are not indicated to be absent of harm nor are they contrasted to similar yet distinctly “harmful” violations.

#### Chimera?

Operationalizations of purity were classified either as a “single operationalization” or as a “chimera operationalization.” Single operationalizations involved sets of scale items or vignettes in which it was clear that all items or vignettes tapped into one singular construct—purity is “x.” For example, the scale items developed by Boer and Fischer (2013) to measure purity all tap respect for God. They explain that their items to assess purity included “general religiosity, religious experiences, and beliefs or the evaluation of religious behaviors, such as church attendance” (p. 1120). Similarly, Casciaro and colleagues (2014) measured purity solely based on pathogen avoidance by having participants complete a task that involved turning word fragments into meaningful words, which could be completed as words related to physical cleansing: “W _ _ H, S H _ _ E R, and S _ _ P” (p. 714).

In contrast, chimera operationalizations involved sets of scale items that collectively tapped into multiple understandings at once, such that, depending on the item or vignette sampled, purity might either be “X,” “Y,” or “Z.” For example, in the set of 9 scale items developed by Graham and colleagues (2009) to measure purity, we found that across the item set at least four different understandings were being measured: (a) maintaining chastity and avoiding sexual taboos, (b) elicitors of disgust, (c) self-control, and (d) maintaining natural order. For example, the item “Chastity is still an important virtue for teenagers today, even if many don’t think it is” is relevant to maintaining chastity, the item “Whether or not someone did something disgusting” pertains to elicitors of disgust, “Whether or not someone did something unnatural or degrading” assesses maintaining natural order, and the item “Whether or not someone was able to control his or her desires” assesses self-control (p. 1044). Similarly, Rozin and colleagues (1999) created multiple vignettes to assess purity. We found that across these different vignettes at least three different understandings were being measured: (a) pathogen avoidance, (b) sexual chastity and avoidance of sexual taboos, and (c) elicitors of disgust. For example, the vignette “A person is eating a piece of rotten meat” assesses both disgust and pathogen avoidance. Alternatively, the vignette “A person (is shaking hands with someone who) has an incestuous relationship” or “A person is hearing about a 70-year-old male who has sex with a 17-year-old female” assesses sexual chastity and avoidance of sexual taboos (p. 578). From these examples, we see multiple understandings represented across different vignettes used to exemplify purity transgressions, with some vignettes simultaneously containing multiple understandings.

#### Results

In Table 2, we summarize our analysis of the operationalizations of purity and provide example operationalizations for each of the 6 cells when crossing Contra-harm (explicit, implicit, stand-alone) and Chimera (single, chimera). Consistent with what we found with respect to the definitions of purity, the majority of purity operations either explicitly (45.9%) or implicitly (26.7%) operationalized the domain of moral purity as immoral behaviors that did not involve harm. Again, only 27.4% of operationalizations conceptualized purity without contrasting with harm. Also consistent with the definitions of purity, most operationalizations of purity were chimeras (95.5%), such that across the different scale items or vignettes composing the purity measure, several understandings were referenced (e.g., pathogen avoidance, acts tied to disgust, chastity, or spiritual integrity).

**Table 2.** Operationalizations of Purity.

	Single operationalization (*n*=6; 4.5%)	Chimera operationalization (*n*=129; 95.5%)
Explicitly contra-harm (*n*=62; 45.9%)	**0 operationalizations**	**62 operationalizations** *“-Whether or not someone did something unnatural or degrading.* ***[natural order]*** *-Whether or not someone was able to control his or her desires* ***[self-control]*** *. . . -People should not do things that are revolting to others*, ***even if no one is harmed***. *-I would call some acts wrong on the grounds that they are unnatural or disgusting.* ***[disgust]****-Chastity is still an important virtue for teenagers today, even if many don’t think it is.* ***[chastity/sexual taboos]*** *-Sign a piece of paper that says “I hereby sell my soul, after my death, to whoever has this piece of paper”.* ***[spiritual integrity]*** *-Attend a performance art piece in which all participants (including you) have to act like animals for 30 minutes, including crawling around naked and urinating on stage* ***[pathogen avoidance]****. . .”* (Graham et al., 2009, p. 1044)
Implicitly contra-harm (*n*=36; 26.7%)	**2 operationalizations** *“* ***Care****/prosocial scales included interpersonal cooperation, altruism, and other prosocial or reverse coded antisocial attitudes, such as* ***violent tendencies****. . .* ***Purity****/religious attitudes included general religiosity, religious experiences, and beliefs or the evaluation of religious behaviors, such as church attendance.* ***[respect for God]****”* (Boer & Fischer, 2013, p. 1120)	**34 operationalizations** *“* ***(Harm/Care)*** *Stick a pin into the palm of a child you don’t know. . .* ***(Purity/Sanctity)*** *Attend a performance art piece in which the actors act like animals for 30min, including crawling around naked* ***[chastity/sexual taboo]*** *and urinating on stage* ***[pathogen avoidance/disgust]***.*”* (Haidt, 2007, p. 999)
Stand-alone (*n*=37; 27.4%)	**4 operationalizations** *“Participants then completed a word-completion task to measure cleansing accessibility [. . .]. The task involved turning word fragments into meaningful words using the first word that came to mind. We provided participants with six-word fragments, three of which (W _ _ H, S H _ _ E R, and S _ _ P)* ***[pathogen avoidance]****”* (Casciaro et al., 2014, p. 714)	**33 operationalizations** *“We asked participants to recall in detail either an ethical or unethical deed from their past and to describe any feelings or emotions they experienced.* ***[general immorality]*** *Then they engaged in a word completion task in which they converted word fragments into meaningful words. Of the six word fragments, three (W _ _ H, SH _ _ ER, and S _ _ P) could be completed as cleansing-related words (wash, shower, and soap) or as unrelated words (e.g., wish, shaker, and step).* ***[pathogen avoidance]****”* (Zhong & Liljenquist, 2006, p. 1451)

*Note*. Shown examples are taken from the most highly cited operationalization available within the category cell.

Our review of the definitions and operationalizations of purity across 158 articles suggests that purity is often understood as a contra-chimera in moral psychology. Of course, there are exceptions to this contra-chimera understanding. Three of 158 articles defined purity as neither contra nor chimera, and 4 of 135 articles operationalized purity as neither contra nor chimera. Nevertheless, we suggest that this analysis provides evidence that many definitions are contra-harm, chimeric, or both. Purity is frequently defined and measured as contra-harm (a harmless wrong), and perhaps as a result of being defined as not-harm, there was substantial variation within and across papers in terms of how purity was defined or measured, manifesting as a chimera of different understandings.

### Contrasting Purity With Other Moral Concepts

**Figure 2.**
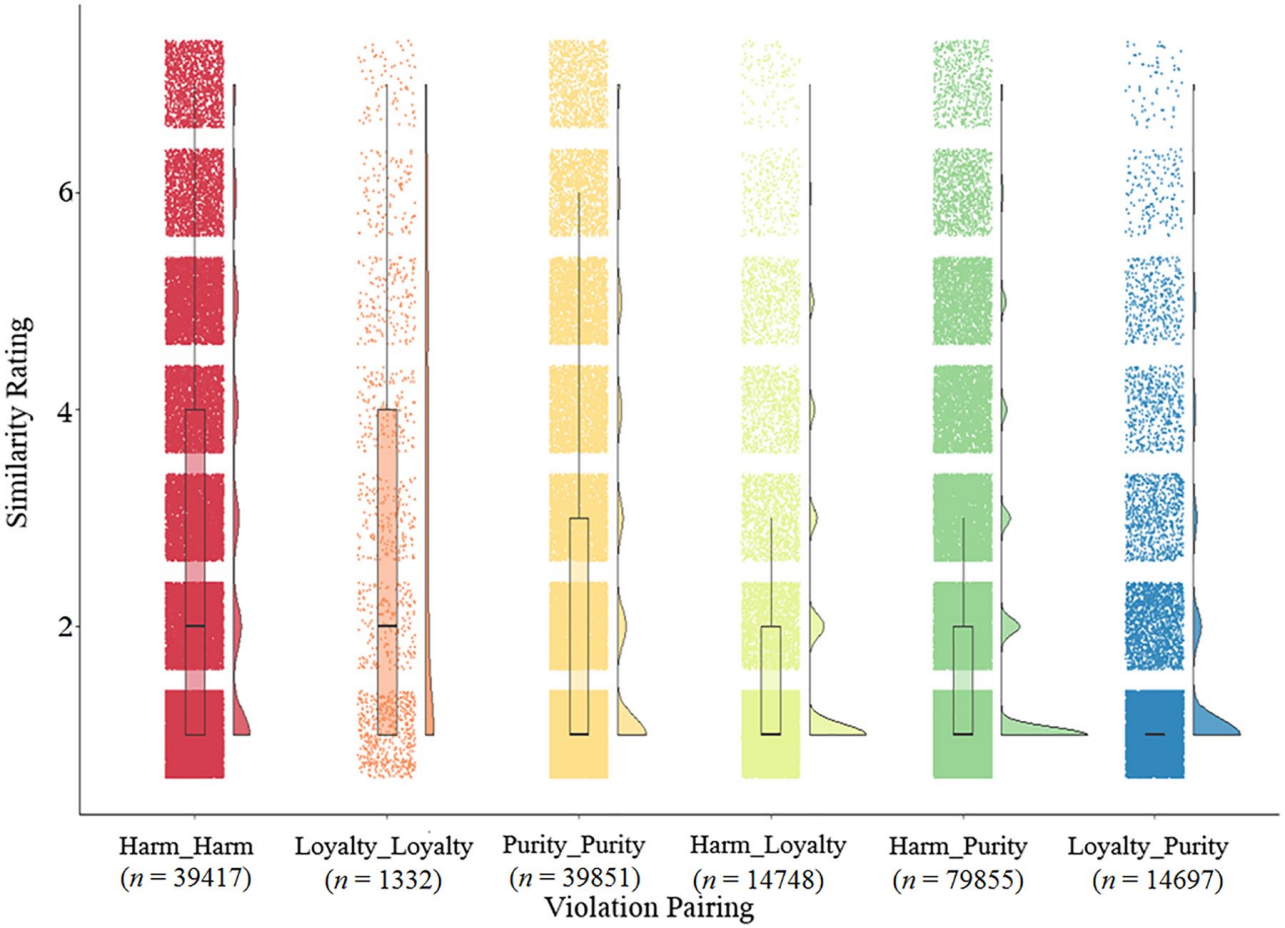
Ratings of similarity between different violation types (taken from DiMaggio et al., 2022—full article preprint can be accessed through the supplement). *Note*. Distribution of similarity ratings within and across the moral domains of harm, purity, and loyalty (*n* =189,900 total ratings). There was significantly more heterogeneity within the purity domain (*m* = 2.27) than within the harm (*m* = 2.76; *p* < .001) and loyalty domains (*m* = 2.89; *p* < .001). We evenly sampled across all pairings, so there were significantly less pairings involving loyalty violations given the small sampling pool.

Purity is a chimera, but is it any more of a chimera than other moral concepts, like harm and loyalty? Two recent studies directly explored the relative coherence of purity compared with two other moral constructs, harm and loyalty (DiMaggio et al., 2022). In their first study, American participants rated the similarity of all the purity, loyalty, and harm violation vignettes typically used in moral psychology literature. As expected, these ratings reveal lower overall similarity (i.e., higher heterogeneity) among purity violation vignettes than among harm and loyalty vignettes, suggesting that purity is an especially heterogeneous moral construct. See Figure 2 taken from DiMaggio and colleagues (2022) for similarity ratings of vignettes within each moral concept as well as across each moral concept.

**Figure 3.**
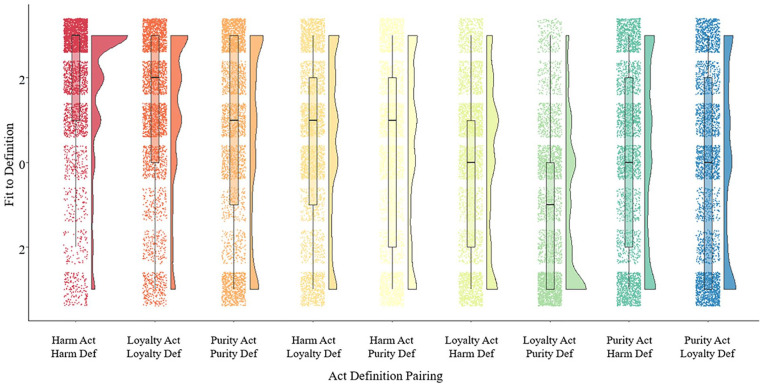
Ratings of fit between domain violations and domain definitions (taken from DiMaggio et al., 2022—full article preprint can be accessed through the supplement). *Note*. Distribution of fit ratings of moral violations to moral definitions within and across the moral domains of harm, purity, and loyalty (*n* = 31,680 total ratings; 3,520 per violation-definition type pairing). Purity violations fit to purity definitions (*m* = .55) significantly worse than harm violations (*m* = 1.91; *p* < .001) and loyalty violations (*m* = 1.17; *p* = .002) fit their respective definitions.

In their second study, DiMaggio and colleagues (2022) explored how well moral vignettes of purity, harm, and loyalty violations corresponded to their respective moral definitions. American participants rated the fit between vignettes of purity, loyalty, and harm violations with overarching definitions of purity, loyalty, and harm. Analyses revealed that, on average, vignettes of purity violations paired with the purity definition were rated as a significantly worse fit than harm vignettes paired with harm descriptions or loyalty vignettes paired with loyalty descriptions. See Figure 3.

In sum, recent empirical work by DiMaggio and colleagues (2022) suggests that purity vignettes are both more heterogenous than harm and loyalty vignettes and show a poorer fit to their definition than harm and loyalty vignettes. In other words, purity is more of a chimera than other moral concepts. One obvious question is what is the nature of this chimera?

## The ~Nine Moral Psychology Understandings of Purity

**Figure 4.**
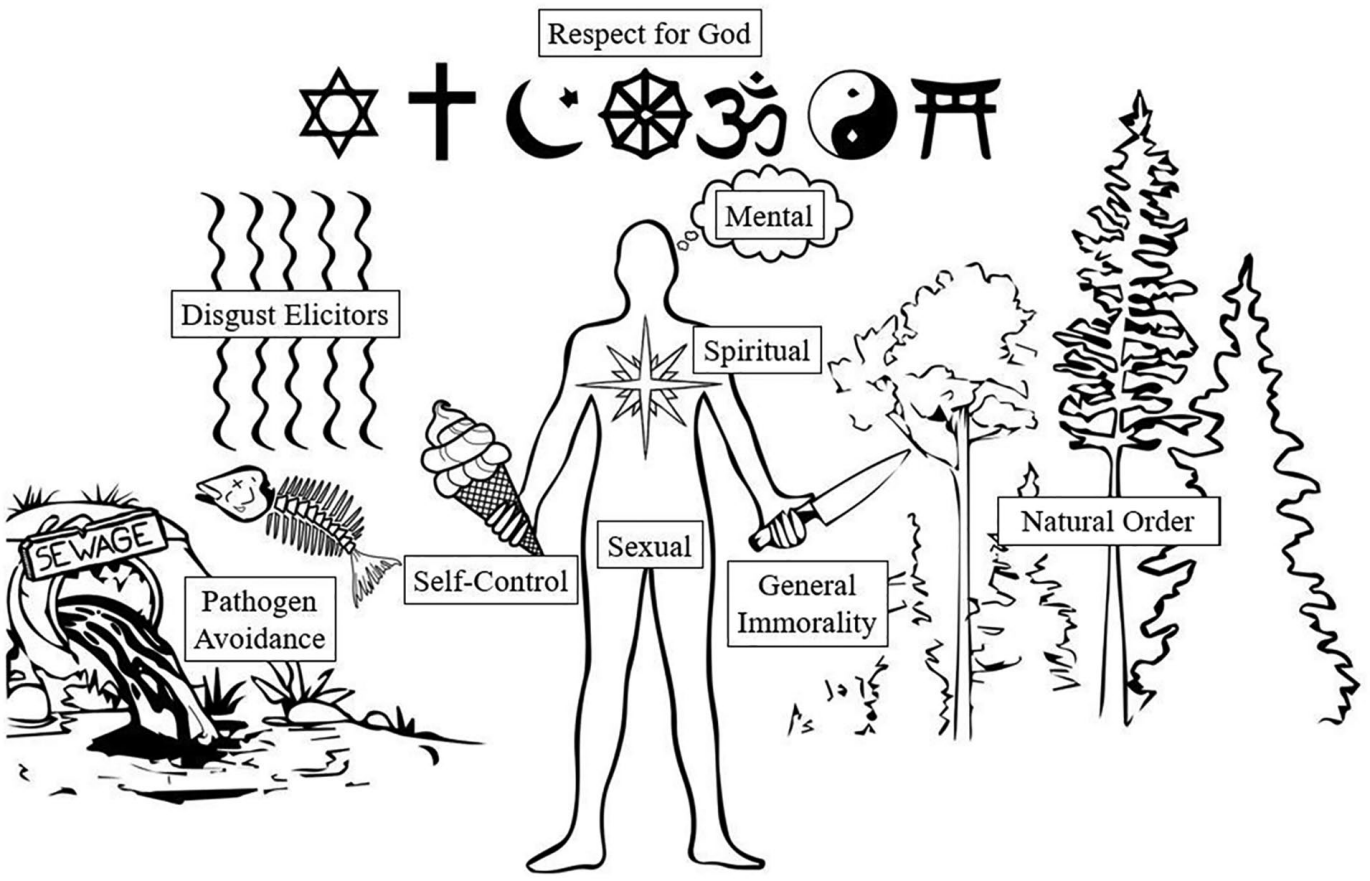
Visual representation of different understandings of purity. Artwork by Ella Gaines.

What are the conceptual components that form the purity chimera from one variant to another? Guided by the historical roots of purity and the development of the construct within moral psychology we inductively identified nine understandings that have commonly been used to define purity. We review each of these understandings below briefly and illustrate them visually in Figure 4. To provide some organization to the nine understandings, we have further ordered them on a continuum stretching from the physical world of bodies and disease to the metaphysical world of souls and deities. Understandings related to mental states and abstract concepts of ethics fall in the middle of this continuum. We also report the prevalence of each understanding within the 158 articles we reviewed (listed in descending order). We summarize all nine understandings in Table 3 and provide examples of how these understandings have emerged both in the definitions and operationalizations of purity.

**Table 3.** Most Common Understandings Used to Categorize Behaviors or Mental/Physical States as Purity.

Understanding used to define purity domain	# of papers	# of citations from papers	Exemplar definition	Exemplar operationalization
Pathogen avoidance	129	21,566	*“The third binding moral foundation—* ***Purity/sanctity—was specifically proposed by*** Haidt and Joseph (2007) ***to be an antipathogen defense system*** *that underlies moral concerns regarding issues of contamination”* (van Leeuwen et al., 2012, p. 431)	Vignette: *“A person is* *** eating a piece of rotten meat****”* (Rozin et al., 1999, p. 578)
Maintaining natural order	87	13,168	*“Purity violations have also been defined as* ***actions that go against the natural order of things****”* (Olatunji et al., 2017, p. 297)	Moral Relevance Question: *“* ***Whether or not someone did something unnatural*** *or degrading”* Moral Judgment Question: *“* ***I would call some acts wrong on the grounds that they are unnatural*** *or disgusting.”* (Graham et al., 2009, p. 1044)
Chastity/sexual taboo	124	18,925	*“One moral domain is purity/sanctity, which promotes appropriateness of social conduct and* ***suppression of carnal impulses, such as lust****”* (Masicampo et al., 2014, p. 2135)	Vignette: *“Phil, who is 18 years old, and his 67-year-old neighbor kiss each other passionately and* ***rub against each other until they climax*** *(Purity)”* (Piazza et al., 2013, p. 715)
Elicitors of disgust	137	21,430	*“virtues of purity and practices regulating food and sex (e.g., Douglas, 1966) bore an* ***obvious relationship to the evolutionary literature on disgust****”* (Graham et al., 2011, p. 368)	Moral Relevance Question: *“* ***Whether or not someone did something disgusting****”* Moral Judgment Question: *“* ***People should not do things that are revolting*** *to others, even if no one is harmed.”* (Graham et al., 2009, p. 1044)
Self-control	40	8,029	*“Purity/sanctity reflects* ***the evolved tendency to place controls on one’s desires****”* (Weber & Federico, 2013, p. 109)	Moral Relevance Question: *“* ***Whether or not someone was able to control his or her desires****”* (Graham et al., 2009, p. 1044)
General immorality	37	5,781	*“* ***Feelings of physical purity seem to embody personal morality and integrity*** *[. . .]. For instance, the mere act of washing one’s hands after committing an immoral action appears to alleviate guilt and other negative feelings [. . .], literally washing away one’s sins.”* (Preston & Ritter, 2012, p. 1365)	Coding Classes: “*People should be pure in what they say (don’t swear/lie/gossip). . .* ***People should be moral individuals***.*”* (Vasquez et al., 2001, p. 118)
Mental purity	24	5,429	*“mental purity (i.e.*, ***the absence of immoral and therefore dangerous thoughts****)”* (Bastian et al., 2015, p. 1070)	Vignette: *“* ***Someone meditates to keep her mind free of impure thoughts****”* (Cannon et al., 2011, p. 327)
Spiritual integrity	79	17,425	*“Divinity/purity violations. . . To decide if an action is wrong, you think about things like sin, the natural order of things*, ***sanctity, and the protection of the soul or the world from degradation and spiritual defilement***.*”* (Rozin et al., 1999, p. 576)	Vignette: *“Sign a piece of paper that says ‘* ***I hereby sell my soul***, *after my death, to whoever has this piece of paper’* (Graham et al., 2009, p. 1045)
Respecting God	81	11,167	*“Divinity/purity violations.* ***In these cases, a person disrespects the sacredness of God****”* (Rozin et al., 1999, p. 576)	Survey Question: *“* ***People should follow religious beliefs****”* (Vasquez et al., 2001, p. 118)

Of course, there is always discretion in how themes are coded, and we are certainly not claiming that there are necessarily 9 eternal and encapsulated domains of purity. Rather, we are carving purity at joints suggested by themes in history and the social psychological literature more broadly. There is a clear precedent for this kind of narrative moral analysis; the genesis of Moral Foundations Theory stems from just such an inductive reading of the literature (Haidt, 2012). We acknowledge that it might be possible to combine some understandings together and arrive at a number less than nine. It may also be possible to split apart some understandings and arrive at a number higher than nine. Regardless of the precise number, the point is that moral psychologists understand the concept of purity in multiple ways—purity is not one “thing.”

Even acknowledging some overlap between themes, it is difficult to argue that self-control, respecting God, and avoiding disease are the very same construct—at least without an *a priori* commitment to “purity” as a single thing. The social psychology literature has distinct literatures on self-control (e.g., Baumeister & Exline, 2000; Masicampo & Baumeister, 2008; Mischel & Ebbesen, 1970), on religious ritual (Beit-Hallahmi & Argyle, 1997; McCauley & Lawson, 2002; Stark, 2001), and on disease avoidance (Curtis et al., 2004; Radley, 1994), suggesting that the field understands these ideas as separate. We also note that these themes can be carved into more pieces. For example, we have combined chastity and avoiding sexual taboos, although one could argue that keeping one’s virginity and not engaging in bizarre bestiality are fundamentally different.

### The Continuum of Purity Understandings (Ordered From Those Rooted in the Physical World vs. the Metaphysical World)

1. **Pathogen avoidance** (129 articles; 81.6% of total articles). Purity has most frequently been described in terms of acts that avoid pathogens or contaminants which may be found in the physical world. This understanding of purity is found in early descriptions of moral purity when Rozin and colleagues (1999) posited that morality emerged as a by-product of the behavioral immune system. From this perspective, acts that expose individuals to biological contaminants are considered impure, and in turn, immoral. For instance, examples of purity violations as biological contamination were offered by Rozin and colleagues: “A person is eating a piece of rotten meat” or “A person is touching a rotten corpse” as “causing impurity or degradation of himself/herself or others” (p. 576, 578). In more recent work, Sheskin and Santos (2012) explicitly defined purity as pathogen avoidance when they described: “Behaviors in the fifth and final domain, the purity domain, focus on avoiding contaminants. The original targets of such behaviors were avoiding ingestion of physical contaminants” (p.12).
2. **Maintaining natural order** (87 articles; 55.0% of total articles). Purity is also linked to the physical environment when it is used to describe objects or behaviors which do not deviate from the natural order. The idea that impure acts are unnatural can also be linked to theories that describe psychological morality as an offshoot of the behavioral immune system (Rozin et al., 1999) in that atypical stimuli in one’s environments are signals of potential physical contaminants or lack of fitness. Formally, the concept of purity has also been operationalized as unnatural behavior with items such as “Someone did something unnatural” (Graham et al., 2009, p. 1044) or “I would call some acts wrong on the grounds that they are unnatural” (Aharoni et al., 2011).
3. **Maintaining chastity and avoiding sexual taboos** (124 articles; 78.5% of total articles). Purity has also frequently been conceptualized as maintaining sexual chastity and avoiding sexual behaviors that deviate from what is normative within one’s cultural context. Within the Old Testament, engagement in improper sexual relations was one way in which people could become ritually impure. In early medical writing, Kellogg also referred to impure acts as involving lustful thoughts and unchaste actions (Kellogg, 1888), while psychoanalytic and psychological folk theories discussed the importance of sexual purity (Agarwal, 1962; Parikh, 1964; White, 1962). Thus, it is not surprising that sexual chastity has featured heavily within the development of the purity concept within moral psychology. For instance, while still conceptualized as “ethics of divinity,” early purity violations offered by Haidt and colleagues (1993)^4^ described siblings kissing each other passionately. Rozin and colleagues (1999) also operationalized impure acts as including incestuous relationships, or sex between two individuals with a large gap in age. Finally, within MFT, purity has also been described as “[. . .] the suppression of humanity’s baser, more carnal instincts” (Graham et al., 2009, p. 1030).
4. **Elicitors of disgust** (137 articles; 86.7% of total articles). Another understanding often referenced in conjunction with purity is disgust. Various forms of purity violations (e.g., those involving pathogen avoidance, or deviant sexual acts) have been described as elicitors of disgust. Thus, because disgust has become so tied to the examples of purity violations, the understanding of eliciting disgust has in and of itself become definitive of moral impurity. For instance, an early description of purity violations offered by Haidt and colleagues (1993) described purity violations as “acts that are disgusting or degrading to one’s spiritual nature” or as “disgusting actions [which] pollute the temple of the body” (p. 614, 615). MFT has also operationalized purity directly as disgust with the item: “Whether or not someone did something disgusting” (Graham et al., 2009, p.1044). The link between purity and disgust developed from the idea that our moral judgment system evolved from our behavioral immune system designed to identify harmful (disgusting) substances in our natural environment (Rozin et al., 1999). Later, MFT offered a more nuanced perspective on this idea, arguing that different moral foundations should be tied to different corresponding emotion systems (i.e., a link between disgust and purity violations and anger and justice violations; Haidt & Graham, 2007).
5. **Self-control** (40 articles; 25.3% of total articles). The concept of purity has also been used in reference to people’s mental states and mental capacities. One prominent example of this has been the conceptualization of purity as self-control. The idea that people who are more capable of controlling their impulses are purer has historical roots. For instance, Kellogg (1888) described purity in relation to self-control when he described the construct as being linked to “*preserving, conscientious efforts* to comply with every requirement of health, purity, morality and the laws of nature” (in “A Source of Crime”). Within the moral psychology literature, early conceptualizations of purity directly operationalized the construct in terms of self-control capacity, “people should be in control of themselves,” to assess moral purity (Vasquez et al., 2001, p. 118). More recent conceptualizations of purity from MFT (e.g., Graham et al., 2009) have also described purity as “suppressing the selfishness often associated with humanity’s carnal nature” (p. 1031) and operationalized purity as “Whether or not someone was able to control his or her desires” (p. 1044).
6. **Not being immoral in a general sense (“general immorality”)** (37 articles; 23.4% of total articles). The term purity has also commonly been used to reference generally immorality or unethical behavior. For instance, Zhong and Liljenquist (2006) manipulated people’s sense of moral purity by asking participants to “recall in detail either an ethical or unethical deed from their past” (p. 1451; see also Earp et al., 2014). The idea that engaging in generally immoral behaviors makes one impure has its roots within moral psychology research examining the “MacBeth Effect,” the idea that immoral acts cause one to become contaminated and in need of washing away their sins (Zhong & Liljenquist, 2006). However, this notion that one must cleanse themselves of their unethical behavior is an ancient concept in many cultures; The Tikopians bathe themselves before rituals to cleanse themselves of their sins before entering the presence of God (Firth, 1960, 1970).
7. **Not thinking immoral thoughts (“mental purity”)** (24 papers; 15.2% of total papers). Purity has also been used in reference to people’s mental states more broadly when it is described as avoiding thinking generally immoral thoughts. Early on, Kellogg (1888) warned people not to “dethrone the mind” (in “A chapter for boys”) while folk theories of purity described “purity of thought” (Agarwal, 1962; Parikh, 1964; White, 1962). Within the domain of moral psychology, Bastian and colleagues (2015) defined purity simply as “the absence of immoral and therefore dangerous thoughts” (p. 1070), while Haidt and colleagues (1993) operationalized purity with the item “People should keep their mind/spirit/thoughts/feelings clean and pure.”
8. **Spiritual integrity** (79 papers; 50.0% of total papers). Purity has not only been described with reference to our physical world (e.g., pathogens or sex) but also our metaphysical (or spiritual) world. Descriptions of purity as spiritual integrity (versus defilement) can be traced back to early descriptions of the construct. When discussing the importance of purity, Kellogg (1888) warned of acts that can “ruin the soul.” The complex moral codes of the Brahmin Indians (Douglas, 1966) also focused on rules to prevent defiling one’s spirit. Within moral psychology, spiritual integrity was also an important component of Haidt and colleagues’ (1993) early description of purity when they described how individuals who value divinity believe that “the self is conceptualized as a spiritual entity striving to avoid pollution and attain purity” (p. 614). Later descriptions of purity by Haidt and Graham (2007) also described how “those who live so that the soul is in charge of the body (chaste, spiritually minded, pious) are seen as elevated and sanctified” (p. 106).
9. **Respecting God** (81 papers; 51.3% of total papers). Beyond the broader understanding of one’s personal spiritual integrity, respect for God specifically has also been closely tied to the purity concept. Early descriptions of purity emerged from Christian sermons where the term reflected Godliness, and the first Mormon, Moses Smith, also described the concept of purity in relation to God (Smith, 1861, p. 33). Within moral psychology, early and highly cited definitions of purity tied the construct to the moral code of divinity, and described purity violations as cases in which “a person disrespects the sacredness of God” (Rozin et al., 1999, p. 576).

Taken together, these nine understandings illustrate many different “genetic” components which may be found within different purity chimeras. Our review of the 158 articles further reveals that, on average, researchers invoke 3.55 (*SE* = .12) of these understandings when defining purity, and invoke 3.40 (*SE* = .11) of these understandings when operationalizing purity. This means that purity researchers typically use a chimeric understanding. Importantly, 116 of the purity papers also contained some discrepancy between the understandings used in definitions versus operationalizations, such as emphasizing spiritual integrity in a definition but using pathogen avoidance in an operationalization. See supplemental materials for more information about the number of understandings across papers and definition-operationalization discrepancies.

## A Summary of the Moral Purity Literature So Far

Our empirical investigation suggests that relative to harm and loyalty, purity is especially heterogenous and disconnected from its theoretical definitions. Our systematic review suggests a reason for these features: the concept of purity in moral psychology is a contra-chimera. The substantial variability in how purity has been defined within and across the 158 moral purity papers in our corpus supports the idea that contra (“negative”) definitions are rather variable because they are unbounded by constraints beyond being not harmful.

Of course, one could argue that there is some intuitive feeling about what purity might be. Our point is that when this vague intuitive feeling is made concrete in the scientific literature, it remains vague, lacking conceptual coherence. Here, we describe more concretely how this lack of coherence is problematic for the scientific trajectory of moral psychology. We focus on four challenges: (a) Problems for theoretical development, (b) Problems for stimulus development, (c) Problems with falsifiability, and (d) Problems for explanation.

### Theoretical Development

The development of theory is a cumulative and iterative process. New theoretical propositions concerning a psychological concept are understood and evaluated within the context of past contributions to the same concept. As a result, for a theory to develop over time, it is vital for the building blocks of the theory to be stable across time. Without this consistency, it is difficult to know whether the theoretical claims made in one article are applicable to the claims in another article, despite both referring to “purity.”

Our review raises questions about the cumulative theoretical understanding of purity. Across the 158 articles we reviewed, purity was defined and operationalized using nine different understandings, which ranged along a continuum of being rooted in tangible physical world phenomena (physical contact with pathogens or physical acts of sex) to mental processes (such as purity of mind or self-control), to metaphysical concepts such as spirits and gods. On average, when articles defined purity, they invoked 3 to 4 of these different understandings. As a result, although our corpus contained 158 “purity” articles, they are difficult to all reconcile with each other, beyond the common thread of lacking obvious dyadic harm. Rather than a growing field of purity *per se*, our corpus likely instead reflects nine smaller subfields.

### Measurement Development

Our review highlights variations in definitions of purity and also variations in operationalizations of purity, which vary both across articles and within articles such that operationalizations are often disconnected from explicit definitions. The variability in how purity is measured—the acts, scenarios, and language that count as “impure”—make interpretations of empirical findings difficult because invariance in measurement is considered to be vital for the cumulative scientific study of any phenomena (Flake & Fried, 2020). Of course, it is challenging to develop a reliable measurement of almost any concept, but when the definitions that give rise to that measurement are so variable, the difficulties are compounded. Perhaps the one unifying thread among purity measurements is that they lack obvious interpersonal harm, but this “contra” criteria further expands the diversity of measurements because the set of these acts is extremely broad.

Other measurement tools use rating scales (Graham et al., 2009)—or word counts (Graham et al., 2009; see also, Sagi & Dehghani, 2014)—that use synonyms of impurity. For example, the popular Moral Foundations Dictionary uses words such as “humble,” “whore,” and “sick” as associations or synonyms for purity (Graham et al., 2009). The problem with these synonym measures is that the language describing purity is as heterogeneous as the concept itself. One study finds that the correlation between synonym-based ratings of impurity and immorality is often greater than .87 (Gray & Keeney, 2015a).

The rich diversity in what acts count as “impure” no doubt reflects the rich diversity in historical understandings of purity and the looseness of language. However, we again highlight that it is possible to agree on a set of core acts, with a set of core characteristics such as in the case of interpersonal harm, where competing theories consistently recognize that child abuse, animal abuse, murder, and assault are harmful. In fact, looking over the many purity articles in which harm was operationalized reveals striking consistency in operationalizations—in contrast to purity.

### Falsifiability

One of the biggest issues with purity’s nature as a chimera is that it can make theories unfalsifiable. If one understanding of purity doesn’t validate a set of theoretical claims, then a researcher can always move to a different understanding of purity. And if someone disproves some purity-related claim, then a researcher can likewise move to a different understanding of purity. The flexibility of being able to choose between nine different understandings of purity and being able to use operationalizations that may or may not accord with those understandings creates researcher degrees of freedom in testing concepts. As the open science movement has elegantly revealed, any increase in researcher degrees of freedom can inflate false positives (Wicherts et al., 2016).

### Explanation

Another issue is that claims about purity often verge on the tautological, which makes it difficult to fundamentally explain concepts. When attempting to explain (or define) a concept *X*, one must invoke other concepts (*Y* & *Z*) and these concepts cannot be the same concept as the one being explained (*X*). You cannot define a concept with itself. Consider how we might explain flight. To explain how birds fly, one could invoke their hollow bones, the shape of their wings, and the lightness of feathers. One could not adequately explain flight by invoking the presence of “being able to fly” or the absence of “flightlessness,” because these concepts are tautological.

Unfortunately, many of purity’s explanations and definitions are tautological, explaining purity-related questions by invoking the concept of purity or impurity. Consider this quote from a classic paper on intuitionism:

Because we all have experience with foods that are easily contaminated, we come to equate purity and cleanliness with goodness in the physical domain. [. . .] experiences in the physical world then form the basis (in many cultures) of conceptual schemes about moral purity—for example, that children start off in a state of purity and innocence but can be corrupted by a single exposure to sex, violence, drugs, homosexuality, or the devil. (Haidt, 2001, p. 825)

This argument suggests that moral purity is a basic—and undefined—state in children (similar to food purity) which is “corrupted” by “contamination”—both synonyms of impurity. Or in other words, kids are pure until they are made impure by exposure to the impure things of sex, violence, homosexuality and the devil. The causal chain here may be rhetorically compelling, but it is less logically compelling, at least compared with something like harm, which is defined through three constitutive elements of intention, causation of damage, and the suffering of the vulnerable (Schein & Gray, 2018). The tautological nature of purity definitions means that when researchers explain immoral judgments of impure actions by identifying the importance of purity, we are left with a tautology. The same is true when researchers use the term “impurity” to refer to general immorality (e.g., sin), and then claim that impurity predicts moral condemnation. What they are really arguing is that immorality predicts immorality.

While any scientific field struggles with questions of theory, measurement, falsifiability, and non-tautological explanation, the diverse historical and scientific understanding of purity makes it difficult to make strong claims about it. Nonetheless, we evaluate four claims about moral purity which have been put forth.

## Claims About Moral Purity

Purity is the lynchpin of much of modern moral psychology. The idea of “harmless wrongs” captured the imagination of moral psychologists and broadened the scope of morality beyond the justice-based theories of Piaget, Kohlberg, and Turiel (Kohlberg, 1981; Piaget, 1931; Turiel, 1983). The field had long accepted that most people considered it immoral to murder, but purity provided more novel questions: Why do people find it loathsome to drive a car once owned by Hitler? Why do most people shudder at the thought of eating Old Yeller when the poor dog’s time finally came to pass?

Purity is so interesting to scholars precisely because of its contra-chimeric nature. Its heterogeneity not only creates an ever-changing set of moral conundrums, but also allows purity to help describe any moral context that researchers want to study, be it the spiritual concerns of different cultures, the rhetoric of political parties, biblical writings, or Kellogg’s musings over the necessities for a healthy mind.

The concept of moral purity has brought with it a set of interesting new questions, and in attempting to answer these questions, researchers have proposed new theoretical frameworks of moral judgment, including the social intuitionist model (Haidt, 2001) and Moral Foundations Theory (Graham et al., 2009). These purity-inspired frameworks have made four bold theoretical claims that build off one another: (a) moral purity is central to real-world moral rhetoric, as demonstrated by examples of moral purity from the Bible, from government speeches, and from small and remote tribes; (b) because purity was found in moral rhetoric, scholars assumed that cultural variations in whether people moralized purity would be the “Rosetta Stone” for unlocking the underpinnings of variations in moral judgment across cultures; (c) because moral purity was considered a unique phenomenon which could vary across cultures, scholars deemed purity its own coherent domain of moral judgment—a domain distinct from other concepts, especially harm; and (d) because purity was to be regarded as a distinct and unitary form of moral judgment, scholars assumed that it must be driven by its own distinct psychological mechanism of disgust.

In part because of the contra-chimeric nature of moral purity, only the first of these four claims is well-founded, with the second perhaps having some evidence. In contrast, the third and fourth claims are generally unsupported. We outline our arguments with respect to each other and these four claims in turn.

### Is Purity Used in Moral Rhetoric?

Much of moral judgment is conveyed through words (Clifford & Jerit, 2013) and sometimes these words are related to purity. In Shweder’s initial interviews, his Indian participants consistently made appeals to the natural order, sanctity, and defilement to explain the immorality of certain acts (Shweder et al., 1987). This language does not just occur in foreign communities. If you were to sit in the pews of a conservative Christian church on Sunday morning, you might hear the pastor rely on terms like “piety,” “sacred,” “clean,” or “profane,” to convey his moral message (Graham et al., 2009). People also invoke concepts of purity, defilement, and sanctity outside of religion to include copyright infringements (Buccafusco & Fagundes, 2015), biomedical advancements (Kass, 1997),^5^ and climate change policy (Severson & Coleman, 2015). For instance, Sachdeva and colleagues (2019) found that purity rhetoric emerges naturally in online environmental campaigns launched on social media in India.

Purity language is also used by political elites on both the left and right. During the 2019 democratic primary debates, South Bend Mayor Pete Buttigieg responded to Senator Elizabeth Warren’s critiques of his hosting a pricey fundraiser as an “unfair purity test” (Levine, 2019). Meanwhile, republican President Donald Trump repeatedly referred to the term impeachment as a “dirty, filthy, disgusting word” (Oprysko, 2019). Disgust language has also been used in discussions of contentious social issues such as gay marriage (Gadarian & van der Vort, 2018). It should be noted, however, that while purity language does occur in political ads, it is used less frequently than other moral content: In one analysis, 45% of political ads used the language of harm, 38% contained loyalty language, 27% authority, 7% liberty, 6% fairness, and only 3% purity (Lipsitz, 2018).

Although our review reveals that the nature of purity is nebulous, it is clear that purity-related moral rhetoric can impact moral attitudes, sometimes further entrenching political opinions, and sometimes leading to attitude change (Day et al., 2014). For example, framing issues like environmentalism in terms of purity can nudge policy attitudes (Kidwell et al., 2013; Rottman et al., 2015), making conservatives appear to be more pro-environmental (Feinberg & Willer, 2013, 2015) but questions remain about what drives this shift. It may just be that some purity-related words suggest that the messaging is coming from a more conservative speaker, and people are more receptive to messages from ingroup members. Purity rhetoric also predicts closeness in social networks (Dehghani et al., 2016), although because purity language is confounded with religious language, these results could simply be explained through religious similarity. Appeals to purity can also backfire, as the language of disgust can be seen as an illegitimate source of moral reason (Gadarian & van der Vort, 2018; Nussbaum, 2010).

It seems clear that people do rely on purity rhetoric in discourse (Jensen, 2015a), but does this really reflect *moral* judgment in a psychological sense? One could object that purity rhetoric is not inherently indicative of moral attitudes. After all, a person could label an act as defiling, unnatural, and impure without making claims about immorality. It is possible that when people condemn certain dietary practices or sexual proclivities, they are merely expressing distaste or disapproval, and not moral censure. As just one example, imagine your elderly grandmother having casual—but safe—sex with other nursing home residents. For many of us, this might seem like a disgusting violation of nature, and plain old wrong, but here “wrong” likely just means unpleasant, or a violation of social expectancy, norms, or rules. “Wrong” is a broader category than “morally wrong,” which is why early moral psychologists differentiated violations of personal preferences (e.g., violating fashion norms) and social conventions (e.g., wearing pajamas to school), from moral violations (e.g., hitting a classmate; Turiel, 1983). According to Turiel’s Social Domain Theory, only moral violations are seen as universally wrong, authority-independent, and deserving of blame and punishment (Turiel et al., 1987; see also Huebner et al., 2010; Skitka & Houston, 2001). When it comes to purity, the key question is whether the “wrongness” of food practices and bathroom etiquette refers to morality or mere social convention.

Research suggests that at least some people, some of the time, view purity violations as universally wrong, independent of authority, and deserving of blame and punishment. In Shweder’s interviews, he asked specifically about Turiel’s signature of immorality (i.e., universality, severity, punishment) and in the Brahmin sample, violations of food, the body, sex, and God met all these criteria (Shweder et al., 1987). A widow eating fish commits a punishable moral violation that is universally wrong even when done in private. Based on this finding, Shweder argued that in non-Western cultures, there was no strict distinction between convention and morality.

Although Shweder took the time to ensure that his participants viewed the violations as immoral, it is unclear whether some of the current purity research is assessing *moral* wrongness or more general wrongness. While some studies of purity do assess the perceived immorality of acts (e.g., Cannon et al., 2011; Helzer & Pizarro, 2011; Young & Saxe, 2011) there is substantial inconsistency in whether purity violations are rated on moral wrongness or general wrongness. For instance, in one of the more common measures of purity, the Sacred Values Scale (Graham & Haidt, 2012), participants list the amount of money that they would require to perform different acts (e.g., acting like an animal for art). While this could indicate absolute moral judgments, it might also simply indicate the strength of a personal preference or convention judgment.

Another open question is how much purity-related rhetoric reflects post hoc rationalization of moral judgments made for other reasons, such as perceived harm (Haidt, 2001). Environmental degradation harms animals, tainted food harms our health, and a faulty vaccine can harm children who receive it. Purity could be a rhetorical flourish that further draws attention to judgments, whether about morality or general badness. But regardless of the causal order of moral rhetoric and moral judgment, it is clear that purity does hold a place in moral rhetoric both historically and today.

### Does Purity Vary Across Cultures?

Purity was originally introduced to moral psychology as a way to describe cultural variation (Shweder et al., 1987). Moral Foundations Theory has pointed to these cultural differences in bolstering the importance of purity. Therefore, we will next turn to the claim that purity varies by culture. There are two different ways to conceptualize the cultural variation of purity: (a) purity is only moralized by some people or (b) everyone moralizes purity, but what is seen as impure and the expansiveness of impurity differs across cultures.

#### Purity for some

One way to explain cultural differences is that only some communities foster purity (Graham et al., 2009). This group difference perspective can be traced to Shweder’s early theorizing (Shweder et al., 1987) and is evident in the early MFT writings, which argues that American culture wars are persistent and nasty (Koleva et al., 2012) because conservatives but not liberals understand moral concerns surrounding purity (Ditto & Koleva, 2011).

However, more recent empirical work directly challenges this stark contrast between liberals and conservatives and suggests that purity concerns are not unique to conservatives (Frimer, 2020). It is easy to think that only conservatives care about purity when one is focused on sexual chastity but looking at liberal core values paints a different picture. Liberals can see impurity in things like vaccinations and environmental pollution (Frimer et al., 2015). In fact, when it comes to environmental concerns, liberals seem to rely on purity more than conservatives. For many liberals, the environment is sacred, and the immorality of pollution cannot be reduced solely to costs born to humans (Rottman, 2014). Therefore, in trying to explain cultural variation in morality, it is not enough to simply appeal to underlying differences in the extent to which purity is moralized.

Liberals and conservatives both care about sanctity, but just through different lenses. It is also the case that other factors, more so than purity might better explain variation in moral judgments between conservatives and liberals. Work by Kugler et al. (2014) suggests that higher levels of authoritarianism actually underlie the greater valuation of purity observed among conservatives versus liberals. Relatedly, it may be that greater preoccupation with specific taboos (e.g., being more offended by the use of expletives) is more common in conservatives versus liberals, and these specific concerns might be confused with broader concerns about purity. In other work, Schein and Gray (2015) found evidence across seven studies that perceptions of harm (more so than purity) explained moral diversity across the political spectrum. Differences in moral judgments between liberals and conservatives hinge upon what they each see as causing harm. Together, this work suggests that perhaps earlier claims about the uniqueness of purity to conservatives was exaggerated, perhaps as a result of using *a priori* conservative moral issues to construct a scale to measure purity (Graham et al., 2011).

#### Purity for all

The second way in which scholars have suggested that purity can vary across cultures is that while everyone has the capacity to moralize purity, people view different acts as impure across cultures. Everyone, liberals and conservatives alike, understands the concept of defilement. Where the disagreement lies is in which acts cause defilement. For conservatives, that might involve sexual acts; for liberals, it might involve pollution (Frimer et al., 2013, 2016). The key to understanding cultural variation is not a simple appeal to purity for some but rather a more nuanced claim about the type of acts labeled as sacred.

Of course, this more nuanced perspective does not explain *why* there is cultural variation in terms of what is found impure. Why is it that liberals care about the sanctity of the environment, and conservatives care about the sanctity of marriage? To answer these questions, one might also need to account for variation in worldview and cultural norms (Asch, 1952; Rai & Fiske, 2011). Furthermore, the “purity for all” perspective does not deny the fact that some cultures might be more prone to purity rhetoric or might be more likely to believe in the antecedents of purity, such as souls. However, if this were the case, then group differences in morality would emerge not from differences in the underlying structure of moral minds, but rather from nuanced differences in cultural worldviews.

One of these important cultural worldviews may be assumptions of vulnerability: What people in each culture believe is vulnerable to harm (Turiel, 2002). If people in a culture believe that food-related acts can condemn someone to hell, it makes sense that they would moralize these harmful acts as impure. Consistent with this idea, Shweder and colleagues (1997) argued that different frameworks of suffering could help explain moral diversity, consistent with the harm-centric Theory of Dyadic Morality (Schein & Gray, 2018).

Although a common consideration—perceived harm—may explain the moral condemnation of purity violations, there is still important pluralism in examples of purity across cultures. Processing your father’s death pollution by eating only vegetables is not the same behavior as removing your shoes before walking into a shrine, which are not the same behaviors as going on a hot-yoga meditation retreat, or signing a contract to demonstrate your commitment to virginity. Nevertheless, these acts may all be moralized based on how much these acts are seen as preventing harm. To again quote Frazer (1922), acts that maintain purity provide “electrical insulators to preserve the spiritual force with which these persons are charged from suffering or inflicting harm by contact with the outer world.” (p. XXI.1) That is, staying pure safeguards people from being harmed by spiritual means.

### Purity Is a Coherent Psychological Construct?

The use of a single term, “purity” suggests that there is a common unifying thread (or single definition) that pulls all these concepts together and distinguishes it from other concepts—especially harm. In other words, the use of the term “purity” suggests that purity has construct validity.

Construct validity requires two things: convergent and divergent validity. First, the things that we label purity must share some important resemblance (convergent validity); we can therefore falsify a definition of purity if the defining characteristics of purity are not present among all behaviors we would expect for people to find impure (Popper, 1963). Second, things we label purity must be different from things that we do not label purity; we can therefore falsify a definition of purity if defining characteristics of purity can be detected among behaviors that are both related and unrelated to purity. Although questions of construct validity are often mistakenly viewed as binary, we acknowledge that this is not always the case. Validity exists upon a continuum, and you can think of this continuum in terms of entitativity. Very high entitative groups are like Orthodox Ashkenazi Jews, who share a common genetic history, similar cultural styles, and a clear common fate. A low-entitative group are people with the name William. Clearly, they all share a common name, but the similarities end there. Some Williams are princes, others are paupers, some children, some old men, some liberal, some conservative. Is purity more like Orthodox Ashkenazi Jews or a group of Williams?

### Convergent Validity

To examine the construct (convergent and divergent) validity of purity, we must look at how this latent construct is measured (in relation to how it is defined). Our systematic review provides tenuous evidence for the *convergent validity* of purity. Belying the singleness of the word “purity,” we revealed nine different understandings of purity (see Table 3) ranging from basic physical concerns shared by all humans (e.g., infection from pathogens) to metaphysical concerns possessed only by some (e.g., entertaining sacrilegious thoughts). Indeed, scholars have lumped acts as diverse as body modifications (Graham & Haidt, 2012), pouring urine over one’s body (Chakroff et al., 2013), masturbating while cuddling a teddy bear (Helzer & Pizarro, 2011), purposefully wearing unmatched clothing (Horberg et al., 2009), and not observing religious holidays (Ritter et al., 2016; Royzman et al., 2014) all into the category of impurity. Descriptively, the majority of the scenarios used to measure purity involve violations of food, sex, body, and God, although some research on purity also includes norms about meticulousness, contact with death, and purity of the mind.

Although defenders of purity claim that all these violations are about maintaining sanctity (a synonym of purity) of the body and the mind, it is not self-evident that all these factors hang together at a psychological level. Of course, this heterogeneity may be part of people’s fascination with purity. Unlike other coherent psychological categories that have a clear prototype (e.g., harm), the exceptional heterogeneity of purity keeps people wondering about its “true” nature—and prevents them from finding a clear answer. In addition to this heterogeneity, purity may be especially interesting to think about because many purity acts seem strange or counterintuitive, especially to cultural outsiders (e.g., not eating chicken to help your father’s soul). We are sympathetic to the fact that purity is fascinating, but raw fascination does not make for rigorous science. Our review found that purity researchers are unable to come to a consensus about how to define purity independent of synonyms and how to best measure the construct.

### Divergent Validity

Is purity separate from related constructs? We suggest that the answer is no. More specifically, we will argue that purity has been confounded with politics, weirdness, religion, and harm. These confounds not only undermine claims about purity as a natural kind but directly undermine conclusions about the explanatory power of purity.

#### Politics

Modern accounts conflate purity with conservativism (Haidt, 2012). The measures used to assess purity sample from conservative concerns (e.g., sexual morality) and fail to account for liberal concerns (e.g., environmental conservation; Janoff-Bulman & Carnes, 2013). As explored earlier, if you include violations of environmental conservation instead of sexual improprieties, liberals are suddenly the ones who care more about purity (Frimer et al., 2015). This conservative-leaning sampling bias directly undermines claims about the predictive power of purity (Koleva et al., 2012). For example, research using the YourMorals.org data found that individual differences in purity concerns predicted foreign policy positions, such as approval of the Iraq War and disapproval of the Kyoto Protocol (Kertzer et al., 2014). This effect was mediated by political affiliation. The authors concluded that differences in purity concerns gave rise to political differences, which in turn influenced public policy opinions.

However, if measurements of purity are already confounded with political ideology, then the argument becomes tautological (differences in politics predict differences in political affiliation). It has long been known that conservatives are more likely to moralize chastity and so using “*chastity is an important and valuable* *virtue*” to measure purity (MFQ-30; Graham et al., 2013) all but guarantees that conservatives will be higher on purity. If instead of chastity, purity was measured by the importance of certain juice cleanses or yoga, (e.g., “Yoga is an important purifying practice”), it would likely reveal that purity is mostly a liberal concern. If purity is conflated with politics, then it is likely that purity is not a distinct psychological construct, but rather, a way of taxonomizing historical differences between liberals and conservatives.

#### Religion

A related issue is that many purity measurements are confounded with religiosity. For example, the MFQ item “Whether or not someone acted in a way that God would approve of” explicitly invokes the approval of God. This makes it impossible for this popular questionnaire to reveal that an atheist might care about purity. Consider again the item “chastity is an important and valuable virtue.” At least in the United States, many religious communities strictly regulate sexuality (Paul, 1994; Sands, 2000). Therefore, differences in the endorsement of a statement like “chastity is an important and valuable virtue,” could reflect differences in purity or differences in religious upbringing. Adding to this ambiguity is the psychometric finding that the purity subscale of the MFT works differently for religious and nonreligious individuals (Davis et al., 2016). Many of the items selected in purity measures reflect a conservative religious ideology, and once again, this conflation undermines conclusions about purity. For example, while there is a strong correlation between moral disapproval of stem-cell research and endorsement of the purity foundation (Koleva et al., 2012), research suggests that it is not purity per se that is doing the explanatory work but rather a common religious factor contributing to both purity and moral opposition to stem cell research (Clifford, Jerit, et al., 2015).

We acknowledge that most researchers would acknowledge that purity can be predicted in part by religion and politics. Again, one of the six questions in the MFQ purity subscale asks specifically about God’s approval. The real question is whether there is something left in “purity” as a standalone concept once we control for all these other factors. In other words, purity may not be “predicted” by politics and religion, but may simply *be* these things—and also potentially weirdness and harm.

#### Weirdness

Another factor undermining conclusions about purity (e.g., that purity functions differently than harm) is that many of these purity violations are conflated with simple weirdness (atypicality) and severity (Gray & Keeney, 2015a). Many of the scenarios in our review used to measure purity (e.g., eating pizza off a corpse; Clifford, Iyengar, et al., 2015) are weirder (i.e., less typical) and less severe than harm judgments (e.g., murder). When studies control for such confounds, cognitive differences between harm and purity disappear (Gray & Keeney, 2015a).

Without any sort of control for weirdness, it is possible that conclusions drawn from the purity vignettes are just about how morality functions when acts are highly counternormative and rare. There is good reason to expect that highly anti-normative and rare behaviors may raise concerns at least about the moral character and future behaviors of individuals. Uhlmann and Zhu (2014) note that

people do have logical reasons for drawing strong character inferences based on acts like having sex with a chicken carcass [. . .] such behaviors are low in attributional ambiguity (Snyder et al., 1979), statistically rare (Ditto & Jemmott, 1989) and therefore high in informational value (Nelson, 2005). (p. 280)

While people may be somewhat dumbfounded when explaining why the act of having sex with a chicken carcass is wrong, they have less difficultly in explaining why the moral character of individuals who engage in such behaviors is questionable (Uhlmann & Zhu, 2014, Experiment 3). Consistent with this idea, Chakroff and colleagues (2017) find that people are more likely to judge individuals who engage in purity violations as being prone to engage in harmful acts in the future, presumably because they are seen as more likely to deviate from any ethical or moral code including those which forbid harm and injustice. Dosage is also found to be less important for purity violations than harm violations—while it is perceived to be worse to starve many goats versus just one, a person only needs to have intercourse with a goat once to be deemed immoral (Rottman & Young, 2019). Again, however, these results may not be because acts like having sex with a goat are impure per say, but because they are so highly atypical and statistically rare that only one violation is needed to signal serious concerns about moral character. However, because experiments such as those by Rottman and Young (2019) explicitly choose *not to* match purity and non-purity actions on atypicality because in their conceptualization “atypicality is a feature of the purity domain” (p. 1153), it is difficult to know whether purity or atypicality is driving these effects.

#### Harm

Consistent with the idea that purity is understood as contra-harm, most of the research offered in support of purity as a distinct construct points to differences between purity and harm (Chakroff et al., 2013; Haidt & Hersh, 2001; Horberg et al., 2009). However, research by some of the authors sheds doubt on the existence of truly harmless moral violations (Schein & Gray, 2018). While researchers can create acts that appear “objectively” harmless, what matters is whether participants are actually perceiving these acts as victimless. In a series of studies, we found that participants automatically perceive suffering in “offensive but harmless” violations (Gray et al., 2014; Schein & Gray, 2018). For example, after reading about a harmless purity violation (e.g., rubbing feces on a Bible), participants rated a child as expressing more suffering. Moreover, research suggests that perceptions of harm mediate the relation between disgust and immorality within the context of harmless impurity acts (Schein et al., 2016). Although researchers specifically designed their acts to be victimless (Haidt & Hersh, 2001), participants still perceived harm in impurity.

In a more direct test of the distinctiveness of purity and harm, participants were asked to rate the harmfulness and impurity of Haidt and colleagues’ harmful and impure moral violations (Graham & Haidt, 2012). While theories about purity often suggest that harm and purity violations should be maximally distinct, ratings of purity and harm were highly correlated (latent correlations, *r* = .89, Schein et al., 2016), and the harmful situations were actually rated as more impure than the impure acts (Schein et al., 2016). Not only does this finding suggest that purity and harm are not as distinct as previously considered, this research also suggests that at times “impurity” might just be a synonym for immorality.

We recognize that reducing all types of moral violations—from murder to eating shellfish—to first-order harms (e.g., suffering children), glosses over interesting facets of descriptive moral diversity. Naming every type of moral violation “harm” is like going to the zoo and calling each creature an “animal.” While accurate, this level of reductionism obscures the beauty of diversity. Nevertheless, labeling each creature as an “animal” is true, especially compared with believing that each creature in the zoo is fundamentally and eternally different (as was long believed about species; Gray, 2014). Understanding all animals as fundamentally similar is also useful if you have pragmatic goals, such as learning veterinary medicine. Likewise, understanding moral violations through a common framework not only reflects how the mind actually seems to work, but also provides a set of tools for many practical questions, such as how to bridge political divides (Schein & Gray, 2015).

Despite the unifying importance of harm in moral cognition, it is true that moral violations vary on a variety of understandings (self vs. other, proscriptive vs. prescriptive; Chakroff et al., 2013; Janoff-Bulman et al., 2009), and it is important to catalog these distinctions. What we try to claim here is that purity may not be quite so distinct from harm. If there is nothing to purity beyond just “the harm that conservatives see in actions that the Bible forbids” then how necessary is a distinct moral psychology of “purity,” versus cataloging variability in the nature of harm.

### Purity Is a Distinct Moral Mechanism?

The strongest claim about purity goes beyond purity as a distinct construct and instead depicts purity as a distinct moral foundation or mechanism (Haidt & Joseph, 2007). Moral Foundations Theory casts purity as a “moral taste bud” (Haidt, 2012) that is triggered in a specific way to a constrained set of purity inputs (e.g., sexual norm violations) and leads to a specific type of moral output (e.g., moral disgust). The benefit of casting purity as a moral mechanism is that mechanisms provide explanatory power. Just as an appeal to taste buds can help explain why ice cream tastes sweet, a purity mechanism in theory could answer why it is that people moralize certain acts; an act is condemned as immoral when it activates the purity foundation.

However, claims about purity as a moral mechanism depend on the existence of a purity construct. For purity to be a unique moral mechanism, there has to be some feature present in all of purity’s inputs and not in other moral inputs that triggers the purity foundation. As an analogy, all sour foods contain at least one type of acid, and sourness cannot be triggered without the presence of this acid. Yet, as purity lacks convergent and divergent validity, and is often defined or measured on the basis of at least nine separate understandings, it is unclear what that feature could be for purity. Putting this concern aside, a cursory review of disgust, the most likely signature of a purity mechanism, strongly suggests that there is not a single mechanism driving the moral condemnation of “purity” concerns.

#### Disgust and purity: A 1:1 mapping?

The central piece of evidence marshaled in support of purity as a distinct moral mechanism is the claim that purity is uniquely connected to disgust, and disgust drives the condemnation of purity violations (Horberg et al., 2009). Providing empirical support for this perspective are studies which show that purity violations evoke disgust more than other moral violations and studies that show that individual differences in disgust sensitivity correspond to the increased moralization of certain purity concerns (Inbar et al., 2009; Wagemans et al., 2018); for a helpful taxonomy of disgust papers, see Pizarro et al. (2011). However, there is also a basis to critique the potential of disgust to be a unifying feature of purity.

What is largely uncontroversial is that people use the rhetoric of disgust when discussing some moral violations (Shweder et al., 1997) and that disgust is at times felt in response to some moral violations. However, that might be the extent of the consensus currently in the field. While some scholars claim that there is a unique 1:1 mapping of disgust to purity (Horberg et al., 2009) where disgust is a unique predictor of purity (Rozin et al., 1999), there is an ongoing debate about whether disgust has a privileged connection to purity violations (Landy & Goodwin, 2015; Pizarro et al., 2011). From the start, there were empirical and theoretical disagreements about the relationship between disgust and purity and the exact nature of disgust and moral judgment is highly debated. In their early theorizing, Rozin and colleagues (1999) argued that moral condemnation of *all* immoral acts should be tied to emotional disgust because our moral judgment system was thought to have evolved from a physical distaste system designed to identify harmful substances in our natural environment. Later, MFT offered a more nuanced perspective, arguing that different moral foundations should be tied to different corresponding emotion systems (i.e., a link between disgust and purity violations and anger and justice violations; Haidt & Graham, 2007). Thus, from MFT we would expect emotional disgust (rather than other emotions such as anger) to increase the condemnation of purity violations.

A comprehensive review of research suggests that there is little evidence for a discrete purity and disgust link (Cameron et al., 2015). At a conceptual level, it is unclear how disgust alone could drive moral concern, as there are many disgusting acts that are not moralized (Pizarro et al., 2011). Nonetheless, many studies have manipulated disgust through dirty desks, disgusting videos, or gross smells and have tested its impact on the moral condemnation of purity and other moral violations (Inbar et al., 2012; Schnall, Haidt, et al., 2008). Some of the earlier studies to empirically manipulate disgust found that disgust elicitors increased the condemnation of *all* types of moral wrongs (Schnall, Benton, & Harvey, 2008; Schnall, Haidt, et al., 2008). For example, exposure to flatulence spray was found to amplify the severity of people’s judgment of moral infractions (Schnall, Haidt et al., 2008), while self-cleansing was found to nullify these effects (Schnall, Benton, & Harvey, 2008). While this study design could be illuminating, contradictory results have engendered confusion. Some scholars find that disgust has a privileged relation with purity (Horberg et al., 2009); others find that incidental disgust elicitors amplify all types of moral judgments (Schnall, Haidt, et al., 2008). More problematic is that the replicability of studies showing evidence for the amplifying effects of disgust on moral judgment has been tenuous (e.g., Johnson et al., 2014; Ugazio et al., 2012). In their meta-analysis, Landy and Goodwin (2015) found that exposure to disgust eliciting stimuli had a significant yet small amplifying effect on condemnation of moral infractions (*d*=0.11, 95% confidence interval [CI]: [0.04, 0.19]), yet this effect became non-significant when accounting for bias to publish significant effects (*d*=–.01, 95% CI [–0.12, 0.10]). It is also important to note that in contrast to what would be predicted by MFT, the amplifying effects of disgust on moral judgment are not specific to purity violations (something we would expect if disgust was a core defining feature of purity violations). In fact, Landy and Goodwin (2015) found that eliciting disgust amplified the moralization of non-purity violations *more* than purity violations. Relatedly, Chapman and Anderson (2014) find that individual differences in sensitivity to physical disgust relate to greater moralization of norm violations outside the purity domain (see also, Jones & Fitness, 2008). In sum, the meta-analysis (Landy & Goodwin, 2015) and the large-scale replication (Johnson et al., 2016) find little evidence that disgust manipulations have any consistent impact on any type of moral violation.

The amplifying effects of disgust for all moral transgressions, even if not distinct to purity, might still support the idea that moral condemnation of all sorts evolved from a physical disgust system (Rozin et al., 1999). However, even if some studies did find that incidental disgust has a consistent impact on moral judgment, it would still be unclear if disgust itself uniquely impacts moral judgments. Many studies on the unique impact of disgust fail to account for important confounds, most especially whether it is any high-arousal negative emotions that can amplify moral condemnation (Cameron et al., 2015; Cheng et al., 2013). For example, Cheng and colleagues (2013) found that among individuals most sensitive to emotional cues, multiple emotions including fear, anger, grief, and disgust elevated moral condemnation of purity violations (e.g., cannibalism) and nonpurity violations (e.g., theft), and these effects were fully mediated by general arousal. Relatedly, Piazza et al. (2013) have found that the experience of anger more so than disgust is uniquely related to the moral condemnation of both harm violations (e.g., kicking a pet dog) and purity violations (e.g., eating a pet dog) and that anger but not disgust was negatively associated with endorsing mitigating circumstances for these transgressions. From these studies, disgust does not seem specific to the condemnation of purity violations, or moral condemnation in general.

Another test of the influence of disgust on purity judgments is a paper on the “Affective Harm Account (AHA)” of moral judgment, in which the authors manipulated embodied feelings of disgust and measured its impact on the moral condemnation of purity violations (Gray et al., 2022). Unlike past papers that increased embodied feelings of disgust (e.g., with fart spray, Schnall et al., 2008), this study *decreased* embodied arousal by administering the drug propranolol, a beta-blocker. Contrary to long-standing hypotheses (Haidt & Hersh, 2001), results revealed that reducing embodied arousal did not directly reduce the moral condemnation of purity violations. However, reducing embodied arousal did indirectly reduce moral judgments through affect-based appraisals—operationalized as people’s perceptions of disgustingness—suggesting one way that purity can connect to moral judgments. It is important to note that perceptions of harm also predicted the moral condemnation of purity violations, again showing that disgust does not uniquely predict moral judgments of purity.

A more basic test for a purity-disgust link is to show correspondence—that disgust is the consequence of purity violation. There is ample evidence to suggest that people report feeling disgusted by norm violations involving sex, God, body, and food (Rozin et al., 1999). However, recent research suggests that even here there is not a 1:1 correspondence between disgust and purity. First, research has found that people also experience anger and contempt in response to purity violations (Gutierrez & Giner-Sorolla, 2007; Royzman et al., 2014). Also, purity violations do not always reliably elicit a disgust reaction: Franchin et al. (2019) and colleagues assessed the spontaneous facial expressions that participants made when listening to recordings of moral transgressions. Both classic purity violations from MFT and violations created by Gray and Keeney (2015a)—which control for atypicality—produced more expressions of anger (as well as more smiling) in participants than expressions of disgust. Research has also found that appraisals of disgust also arise from other types of moral violations; people feel disgusted when a moral violation indicates a corrupt character, even if the act is unrelated to “purity” (Giner-Sorolla & Chapman, 2017).

In sum, the evidence of a distinct psychological mechanism for moral purity—disgust—is not well founded. The existing empirical evidence raises at least three important concerns about the nature of the connection between disgust (or disgust sensitivity) and moral condemnation of purity concerns: First, disgust does not seem to be uniquely linked to purity violations but is rather associated with the moralization of all infractions (Chapman & Anderson, 2014; Landy & Goodwin, 2015). Second, moral condemnation of purity violations are not only heightened by disgust but also by general negative arousal (Cheng et al., 2013). And third, there is some evidence that anger more than disgust appears to be tied to moral condemnation, even in the context of purity violations (Piazza et al., 2013).

## Taking Stock and Looking Forward: Alternatives to Purity

The study of purity has done much for moral psychology. It has increased our recognition of moral diversity, inspired the intuitionist perspective, and propelled researchers to consider whether individuals moralize acts that lack obvious, concrete, and immediate interpersonal harm. However, the desire to try and apply one convenient label and process (purity perceptions) to explain a diverse array of moral judgments across different cultures and contexts has created theoretical confusion. Purity has emerged as a contra-chimera: The set of all things that do *not* involve obvious and immediate harm. As such, purity is a grab bag of moral concerns without a unifying theme beyond maintaining sanctity and avoiding contamination—synonyms and antonyms that render the overarching understanding of purity tautological.

It is clear that there is cultural diversity, moral intuitions, and important considerations beyond direct physical harm. The idea of purity is not necessary for any of these claims; you can still have cultural diversity, intuitionism, and considerations beyond concrete harm even without purity. However, many individual understandings of purity are important, and so we suggest that moral psychology should identify the various psychological processes reflected in different variations of the purity chimera. In pulling out these “sub-sets” of moral purity, we may find that rather than there being one important “purity” concept there are many different stand-alone concepts that each possess construct validity and falsifiability. Here, we focus on four concepts: (a) Pathogen Avoidance, (b) Cultural Assumptions, (c) Higher-Order Harms, and (d) Signals of Cooperation.

### Dissecting the Chimera

#### Pathogen avoidance

One possibility is that some subset of the actions which have been labeled as impure are those that activate our behavioral immune system (Schaller & Park, 2011). There is a clear evolutionary need to avoid pathogens in the environment. To better navigate pathogens, some scholars postulate the existence of a behavioral immune system: A system of psychological mechanisms geared toward detecting and avoiding pathogens (Schaller & Park, 2011). Pathogen avoidance could explain why people moralize contact with death, the consumption of disease-carrying foods, and sexual acts that could result in infections (Graham et al., 2013). All these acts involve clear disease-related risks, and strong moral norms are a helpful way to culturally transmit pathogen-avoiding behaviors.

We doubt, however, that an appeal to pathogens alone can explain all of purity. Many acts that involve pathogens are not moralized (e.g., children playing in dirt), and recent research suggests that the feeling of queasiness/revulsion associated with pathogen avoidance is not present in divinity concerns (Piazza et al., 2017). Although researchers have appealed to pathogen avoidance to explain the moral condemnation of concerns ostensibly unrelated to pathogens (e.g., xenophobia, order, divinity; Haidt et al., 1997), pathogen avoidance might—at best—capture only a subset of purity concerns.

#### Cultural assumptions

Another possibility is that purity language is used in reference to behaviors that are tied to a group’s specific cultural assumptions and norms about the ideal, valued, or right way in which group members *should* behave (Rai & Fiske, 2011). In this context, the “purity” of a person’s behavior is reflective of whether that person conforms to and abides by what is valued and expected within their group (i.e., the group’s cultural assumptions). For instance, consider our earlier example of the democratic primary candidate who described being asked to pass a liberal “purity test”: Here “purity” is used as a proxy to describe the extent to which the candidate conformed to the normative behaviors valued within “liberal” culture. To be “pure” in this sense is to be a prototypical member of one’s cultural group who conforms to what the culture assumes is ideal.

From a cultural assumptions perspective, to act impurely is to contaminate the shared set of values and norms that members within a specific cultural group value and idealize. For instance, Rai and Fiske (2011) described how “[. . .] ‘impure’ moral acts (Haidt et al., 1993), such as odd sexual fetishes, will be judged negatively and punished because they pollute and endanger the cohesion of the social group” (p. 66). If purity is tied to the cultural assumptions (identity) of a group, then we might expect that members within the same group (with the same cultural assumptions) have a more similar construal of what purity means for them versus when compared with members from different cultural backgrounds. Supporting this idea empirically, Dehghani and colleagues (2016) have provided evidence of the social binding properties of purity language: In an analysis of more than 700,000 posts on Twitter, purity-based rhetoric (but not rhetoric based in the other MFT foundations) predicted network proximity between two people. However, the apparent association between purity language and social matching on Twitter could be due to purity’s confound with religion and politics—which we know drives people’s social connections.

Conceptualizing purity as behaviors that align with cultural assumptions can help explain the high degree of cross-cultural variance with respect to *what* behaviors people find impure. If purity violations are deviations from relatively specific (and perhaps somewhat arbitrary) cultural assumptions, then we should expect variation in purity violations across cultures where these assumptions vary. For instance, as Rai and Fiske (2011) note: “in some cultures, sexual relations with your older brother’s wife, or with your father’s brother’s daughter, are incest; in other cultures, where [. . .] relationships are constituted differently, sexual relations or marriage among these kin are prescribed.” (p. 66).

A cultural assumptions account of purity can also explain why purity seems to be valued *more* in some groups than others, at least when purity values are assessed with one scale that might be anchored to one specific set of cultural values. For instance, why are the “purity violations” of MFT moralized more strongly among conservatives versus liberals? It is possible that because many of the MFT purity violations pertain to deviant sexual behaviors or religious infractions, it is confounded with what conservatives (more than liberals) assume goes against the cultural assumptions of their group. Liberals, who tend to be more open to sexual exploration and movement away from the Church, may have different culturally based assumptions with respect to the sexual behaviors described within the MFT purity scale. Thus, it is possible that liberals moralize MFT purity behaviors less than conservatives not because they care less about “purity” in general, but because they care less about the specific culturally relevant norms and values included in the MFT.

#### Higher order harms

The acts that psychologists label as impure may also reflect abstract moral mandates about harm (tied to a group’s cultural assumptions) that do not involve direct physical harm. Violations of purity can be thought of as abstract/indirect harms or what might be called “*higher-order harms*.’’ While not directly, objectively harmful themselves, purity concerns are abstract norms that depend on the combination of a particular worldview scaffolded on top of harm. It is easy to teach a child about the immorality of concrete first-order harms (e.g., don’t bite your sister), as children already have strong empathy inclinations, an aversion to suffering, and an ability to detect other’s intentions (Eisenberg et al., 2006; Hamlin et al., 2007; Vaish et al., 2010). It is much harder for a child to acquire more abstract moral principles, like dietary practices or religious rituals. To understand why it is wrong to eat pork, the child first must adapt a particular worldview. This required socialization is why purity norms emerge later in childhood (Jensen, 2015b)—toddlers who are averse to witnessing suffering are happy to touch poop—and why purity norms are not as universal as first-order harms (and therefore might seem strange to outsiders). Purity violations require contextualization within a particular worldview.

Initial research suggests that this socialization process is built upon harm. While a child might initially explain the immorality of stealing in terms of autonomy (stealing a toy harms the other child), cultural religious socialization might later lead the child to explain the same act in terms of divinity (stealing violates God’s will). Thus, the transmission of divinity norms can involve associating God’s will with care and compassion (DiBianca Fasoli, 2018). It could also occur through associating a purity violation with direct harm (e.g., a fairy dies every time you don’t clean your room). Children’s natural inclination for helping others and preventing harm can be used as the “raw ingredients” to teach children about God’s will and more abstract moral principles. What prepares people to moralize purity concerns is an innate concern with preventing harm paired with abstract thinking that is situated within a particular worldview.

Conceptualizing purity as higher-order harms also helps us understand why there tends to be larger cross-cultural variation in agreement as to whether “purity” violations (e.g., eating pork) are immoral versus the low-level cross-cultural variation we see with respect to whether prototypical dyadic-harm violations (e.g., rape) are immoral. The Theory of Dyadic Morality (Schein & Gray, 2018) predicts that moral judgments revolve around a *fuzzy cognitive template* of harm defined to the perceiver as an intentional agent causing damage to a vulnerable patient. Importantly, this is a template of *perceived* harm, which means that different people and cultures can see different acts as more or less harmful, based on their cultural assumptions. For example, cultures that believe in an afterlife can see behaviors that disrespect the dead as harmful and thus judge them as immoral. The malleability of perceived harm with respect to cultural assumptions allows for moral diversity by providing for harm diversity. In other words, morality can be pluralistic—with different understandings across places and people—because harm is also pluralistic.

This rich understanding of perceived harm stands in contrast to the idea that harm is restricted to a specific module that is only “triggered” by witnessing direct physical suffering like child abuse. Of course, the direct physical suffering of child abuse is a paradigmatic example of “dyadic harm” consisting of a clear intentional agent causing obvious damage to a vulnerable patient, and these examples are typically seen as immoral. Although many theories agree that paradigmatic examples of harm like child abuse lie at the center of people’s understanding of harm, dyadic morality argues that people view other acts as harmful *to some degree*, depending on how well it matches their dyadic template.

Moral psychology often assumes that harm is either present or absent, but a dyadic template means that there is a *gradient* of harmfulness within people’s minds, based on how well an act seems to match their dyadic template. The potential harmfulness of acts spans a continuum of “dyadicness” which varies in terms of how much an act intuitively seems to reflect an intentional agent causing damage to the vulnerable patient. At the center of the fuzzy cognitive template are behaviors with maximum dyadicness (and therefore maximum immorality), such as rape, abuse, and murder. On the fuzzy fringes of the cognitive template are behaviors with less dyadicness, such as masturbation or eating genetically modified organisms (GMOs) which might not be deemed immoral by everyone. We suggest that these “fringe behaviors” reflect more abstract higher order harms based on a group’s cultural assumptions. Purity violations are “higher order harms” because they need additional elaboration within a cultural world view to be understood as somewhat harmful, and therefore as somewhat immoral.

**Figure 5.**
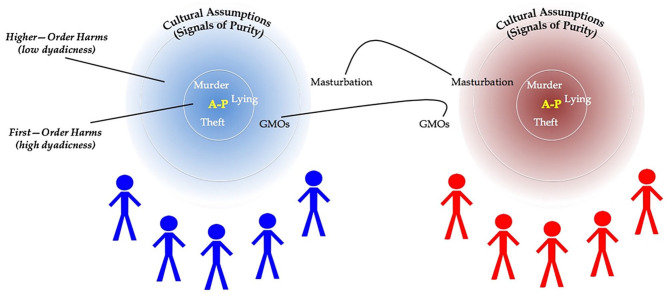
Purity violations can be understood as abstract higher order harms, the violation of cultural values causing subsequent harm. *Note*. First-order harms (e.g., murder, lying, and theft) have relatively high dyadicness (immorality) and fall within the fuzzy cognitive template of harm across cultures. Higher order harms (e.g., GMOs, Masturbation) have relatively low dyadicness and fall within the fuzzy cognitive template of harm of some cultures but outside the template of other cultures. Higher order harms are based on “cultural assumptions” of right and wrong which signal “purity” of membership to one’s cultural group. GMO = genetically modified organism.

Critically, while higher order harms will seldom make it into the very center of the fuzzy cognitive template that is reserved for first-order harms (regardless of culture), their proximity to the center might very well vary based on the cultural assumptions of the group. For instance, consider the fuzzy cognitive templates based on liberal and conservative assumptions of morality: For liberals, GMOs may be found closer to the center of the template (closer to maximum dyadicness) while masturbation might be relegated to outside the fuzzy template (minimum dyadicness). On the contrary, for conservatives, masturbation might be placed closer to the center while GMOs are relegated to the outside. Importantly, because higher-order harms are tied to the group’s distinct cultural assumptions of right and wrong, moralization of these higher-order harms sends an important signal to other group members as to whether one is “pure” in terms of their alignment and conformity to important group values (Figure 5 provides a visual depiction of this idea).

We argue that purity has often been used as a convenient label to describe culturally variable moral transgressions which may be within the fuzzy cognitive template of harm for some cultures (but not others), thus giving purity violations the illusion of being harmless wrongs. However, there is good evidence that purity violations are higher-order harms that fall within the fuzzy cognitive template of harm for some groups, and this challenges the very idea of harmless wrongs.

Supporting the idea of purity violations as higher-order harms is the reliable empirical link between intuitive perceived wrongness and intuitive perceived harmfulness of purity violations (Schein et al., 2016). To the extent that someone sees a purity violation as immoral, they see it as harmful. Often this harm is indirect (Gray et al., 2014). For example, when gay-rights opponents see gay marriage as immoral, it is not often because they see it as immediately harming children (Bryant, 1977). Rather, opponents see gay marriage as destroying the general structure of the heterosexual family, which they see as providing a stable basis for teaching kids morality. Without this stable family, they see children as being unable to learn morality, thereby starting a nationwide slide into evil and anarchy. More broadly, violating purity creates higher-order harms like destroying the cohesion of social groups (Graham & Haidt, 2010) and undermining social order (Janoff-Bulman & Carnes, 2013), which then result in more concrete harms. Indeed, in a qualitative study where participants were asked to identify the victims of apparently harmless purity violations, they frequently pointed to this group-level damage (Gray et al., 2014). It is then just a short mental jump from these higher-order harms to more concrete lower order harms.

#### Signals of cooperation (Character)

Also compatible with the idea that purity judgments are tied to culturally held assumptions of right and wrong, a sub-set of purity violations may reflect behaviors that are used by group members as indicators for whether people would make reliable and cooperative group members (Chakroff & Young, 2015; Giner-Sorolla & Chapman, 2017; Pizarro & Tannenbaum, 2011). Because purity infractions (at least those described within MFT) are often highly atypical, and are thus statistically rare (Ditto & Jemmott, 1989) and low in attributional ambiguity (Snyder et al., 1979), they can provide high informational value about the character of the actor (Nelson, 2005; see Uhlmann et al., 2015 for review). For instance, we feel disgusted at the thought of a man scrounging through the trash to find women’s discarded underwear (Clifford, Iyengar, et al., 2015), or eating the family’s pet (Schnall, Haidt, et al., 2008), because these acts suggest a severe character deficit (Chakroff et al., 2017). Thus, we assume that a person who commits these acts has desires that are beyond the scope of normal human feelings and are dangerous to society. People are more likely to think that people who commit purity violations will engage in harmful behaviors in the future (Chakroff et al., 2017). Although we might never commit murder ourselves, we can understand the situational pressures and thoughts that might lead a person to kill their adulterous spouse. Having sex with a corpse, however, is so abnormal that it suggests a fundamental deficit in moral character (Chakroff & Young, 2015). Purity and harm feel different because violations of purity signal a fundamental break from basic social norms.

### Best Practices for Purity Research

The term “purity” provides an intuitive label to taxonomize a segment of moral diversity. However, labels can be dangerous, because they encourage reification—casting abstract ideas as distinct, concrete entities that all emerge from the same mechanism. Instead of treating purity as a unique moral dimension, treating purity as a cluster of moral concerns opens up the possibility that there are numerous mechanisms that have given rise to purity judgments, that are not themselves unique to purity. The possibilities offered earlier are not meant to be an exhaustive list and more research is clearly needed to disambiguate purity further.

We offer three practical ways in which researchers can address this ambiguity in future research: (a) Ensuring purity research examines only one construct at a time; (b) dissociating purity from atypicality; and (c) being cognizant of what cultural assumptions are imbedded in purity violations.

#### Ensuring purity research examines only one construct at a time

Scholars often design purity vignettes with the goal of representing harmlessness. Because of this contra-harm focus, scenarios often combine several different understandings of purity. For instance, the act of smearing feces on the Bible likely conflates pathogen avoidance with higher order harm to “God.” Similarly, the act of paying sexual compliments to your teacher conflates sexual purity with concerns about respect for authority figures (a completely distinct moral concern from purity within MFT). By making “double-barreled” purity vignettes in this way, it will be impossible for researchers to pinpoint what precise features of a purity violation are what impacts people’s moral judgment. Going forward, researchers need to choose more carefully what psychological concept they want to capture with any one item. Importantly, doing this will be accomplished more easily by trying to measure one clearly defined psychological single-definition concept, rather than a highly variable contra-chimera concept.

#### Dissociating purity from atypicality

Purity researchers also sometimes purposefully conflate purity violations with atypicality or weirdness (e.g., Rottman & Young, 2019) because they operationalize atypicality as being one core defining feature of purity. Yet, atypicality in and of it self may have important implications for how people judge moral character (e.g., Uhlmann et al., 2015; Uhlmann & Zhu, 2014). The conflation of atypicality with purity is problematic because it becomes difficult to know whether it is the specific content of a purity violation (beyond its atypicality) that drives the interesting effects which have been associated with purity violations (e.g., the impact of purity violations on character judgment, differences in dose-response of purity violations relative to harm violations, or the signaling value of purity violations for future harmful behaviors). Previous research has already shown that purity violations need not necessarily be atypical (e.g., Franchin et al., 2019; Gray & Keeney, 2015a). Thus, a fruitful avenue for future research will be to test whether interesting purity effects such as those noted by Uhlmann and Zhu (2014), Rottman and Young (2019), or Chakroff and colleagues (2017) replicate when purity is experimentally disentangled from atypicality.

#### Being cognizant of cultural assumptions that constrain generalizability

Cultural assumptions matter when it comes to understanding morality, especially purity. Although research on purity includes participants from the United States, South America, and India—more diversity than in many fields of psychology—the definitions, operationalizations, and theoretical conclusions drawn from studies are all filtered through the cultural lens of moral psychologists, who are largely Western and White. This presents a *constraint on the generalizability* of purity research, because the cultures studied may not share the same assumptions and/or priorities as the researchers (Roberts et al., 2020).

Many purity violations used by researchers are entangled with cultural assumptions, representing concrete threats to politically conservative or Judeo-Christian religious values. On this basis, some scholars have concluded that conservatives are cognitively hardwired to care more about purity than liberals (Haidt, 2012). However, we know that liberals can care very much about purity violations when they are tied to threats to sacred values of their own social ingroup (e.g., environmental concerns; Frimer et al., 2015). Going forward, it is important for purity researchers to remain vigilant to the full cultural understanding of both themselves and their participants. By doing so they can temper the claims they make about deep cross-cultural differences about purity or moral cognition. There are obviously descriptive differences in the moral frames that people emphasize across cultures (Rai & Fiske, 2011), but these may not reflect differences about purity *per se*, but rather differences about the specific scenarios used by specific researchers.

It is also important to recognize that different groups make different assumptions about what is harmful (Turiel et al., 1991), and these assumptions of harm powerfully shape moral judgments (Gray et al., 2022). If researchers want to understand what is impure and immoral across cultures, they need to fully appreciate the cultural assumptions of those cultures. The last 20 years of confusion about purity might have been avoided if researchers realized that impure acts that seem harmless to researchers (e.g., a son eating chicken after a funeral) were still seen as harmful to people within the cultures they are studying (e.g., Brahmin Indians).

##### Statement of positionality

Speaking of assumptions, we acknowledge our own positionality. We, the authors, are all white people from Canada and the United States. In this sense, we approach moral psychology with a similar identity to much of the field, and with a similar perspective that mostly focuses on judgments made by white Americans about scenarios involving “raceless genderless strangers” (Hester & Gray, 2020).

Despite sharing the same identity position with much of the field, we have a very different theoretical position from many moral psychologists. The current field is dominated by Moral Foundations Theory, and it’s our position—both from our own studies and from carefully reading work in MFT—that the empirical truth and practical utility of this theory has been far overstated.

We believe that an alternative theory—the Theory of Dyadic Morality—better reflects the nature of the moral mind. We acknowledge that the Theory of Dyadic Morality also has its roots in the theoretical perspectives of White/Western authors (e.g., Turiel, Kohlberg). Yet as we describe, the cognitive processes described by dyadic morality can be integrated with pluralistic conceptions of morality, including theories that provide interesting taxonomies of descriptive moral differences (e.g., Janoff-Bulman & Carnes, 2013; Rai & Fiske, 2011; Shweder et al., 1997). Although we are advocates for the Theory of Dyadic Morality, we hope this review about purity will be interesting to all moral psychologists, no matter their theoretical leanings.

## Conclusion

Purity is an ancient concept that has moved from historical religious rhetoric to modern moral psychology. Many things have changed in this leap—Dr. Kellogg would never have imagined a scientific discipline catalyzed by an example of loving incest—but purity still seems to be a heterogeneous concept with diverse understandings. This diversity makes purity an exciting topic to study, but our review suggests that purity lacks a common core, beyond involving acts that are less-than-obviously harmful. Without a consistent and nontautological understanding of purity, it is difficult to argue that purity is a unique and distinct construct, and it is impossible to argue for a mental mechanism dedicated to purity. It is clear, however, that purity is featured in moral rhetoric and can help shed light on cultural differences. Moving forward, we suggest that the field should unpack the richness of purity and explore its many understandings. Moral psychology should also better recognize how perceptions of harm and judgments of wrongness go hand-in-hand, which argues against the popular idea of purity violations as “harmless wrongs.” Most importantly, when conducting research on purity, we should consider not only what purity isn’t but what it really *is*.

## Notes

1 Kellogg believed that eating his cornflakes every morning would promote health and stave off sexual urges.

2 One could technically argue that a positive set could be infinite, such as the set of all positive integers. However, the negative set of numbers that are not positive integers is *more* infinite. For a discussion of this idea, see Georg Cantor’s discussion of infinite sets (Cantor, 1874).

3 Our search yielded 1,033 papers initially. The majority of these papers investigated a distinct concept of purity unrelated to moral purity, such as pharmacology (e.g., Parrott, 2004) and color perception (e.g., Pridmore, 2007), and were not included. We also excluded articles that included purity as a measure but did not offer a definition or operationalization of moral purity. For example, some articles include purity as a measure when applying Moral Foundations Theory (of which purity is one moral foundation) without giving any definition of what purity is and without analyzing the purity domain in their results. Instead, they used purity as a sub-measure of moral foundations, often combining it with other “binding” foundations (i.e., loyalty and authority) and did not draw any conclusions about the purity domain specifically (e.g., Van Leeuwen & Park, 2009).

4 Because purity was still referred to as “ethics of divinity,” Haidt and colleagues (1993) did not appear in our purity search, but it is included here in the subdomain descriptions to highlight the development of the domain.

5 Although purity language is used to describe stem-cell research, political science research suggests that purity language occurs relatively rarely in public discourse (Clifford & Jerit, 2013).
